# The RNA-binding protein SERBP1 functions as a novel oncogenic factor in glioblastoma by bridging cancer metabolism and epigenetic regulation

**DOI:** 10.1186/s13059-020-02115-y

**Published:** 2020-08-06

**Authors:** Adam Kosti, Patricia Rosa de Araujo, Wei-Qing Li, Gabriela D. A. Guardia, Jennifer Chiou, Caihong Yi, Debashish Ray, Fabiana Meliso, Yi-Ming Li, Talia Delambre, Mei Qiao, Suzanne S. Burns, Franziska K. Lorbeer, Fanny Georgi, Markus Flosbach, Sarah Klinnert, Anne Jenseit, Xiufen Lei, Carolina Romero Sandoval, Kevin Ha, Hong Zheng, Renu Pandey, Aleksandra Gruslova, Yogesh K. Gupta, Andrew Brenner, Erzsebet Kokovay, Timothy R. Hughes, Quaid D. Morris, Pedro A. F. Galante, Stefano Tiziani, Luiz O. F. Penalva

**Affiliations:** 1 Children’s Cancer Research Institute, UT Health San Antonio, San Antonio, TX 78229 USA; 2 Department of Cell Systems and Anatomy, UT Health San Antonio, San Antonio, TX 78229 USA; 3 Shanghai Changzheng Hospital, Second Military Medical University, Shanghai, China; 4 Centro de Oncologia Molecular, Hospital Sírio-Libanês, São Paulo, São Paulo 01309-060 Brazil; 5 Department of Nutritional Sciences, Dell Pediatric Research Institute, Dell Medical School, The University of Texas at Austin, Austin, TX 78712 USA; 6 Donnelly Centre, University of Toronto, Toronto, ON M5S 3E1 Canada; 7 Department of Medicine, UT Health San Antonio, San Antonio, TX 78229 USA; 8 Mays Cancer Center, UT Health San Antonio, San Antonio, TX 78229 USA; 9 Department of Molecular Genetics, University of Toronto, Toronto, ON M5S 1A8 Canada; 10 Canadian Institute for Advanced Research, MaRS Centre, West Tower, 661 University Avenue, Suite 505, Toronto, ON M5G 1M1 Canada; 11 Department of Computer Science, University of Toronto, Toronto, ON M5T 3A1 Canada

**Keywords:** GBM, SERBP1, RNA-binding protein, Cancer metabolism, One carbon cycle, Epigenetic regulation

## Abstract

**Background:**

RNA-binding proteins (RBPs) function as master regulators of gene expression. Alterations in RBP expression and function are often observed in cancer and influence critical pathways implicated in tumor initiation and growth. Identification and characterization of oncogenic RBPs and their regulatory networks provide new opportunities for targeted therapy.

**Results:**

We identify the RNA-binding protein SERBP1 as a novel regulator of glioblastoma (GBM) development. High SERBP1 expression is prevalent in GBMs and correlates with poor patient survival and poor response to chemo- and radiotherapy. SERBP1 knockdown causes delay in tumor growth and impacts cancer-relevant phenotypes in GBM and glioma stem cell lines. RNAcompete identifies a GC-rich region as SERBP1-binding motif; subsequent genomic and functional analyses establish SERBP1 regulation role in metabolic routes preferentially used by cancer cells. An important consequence of these functions is SERBP1 impact on methionine production. SERBP1 knockdown decreases methionine levels causing a subsequent reduction in histone methylation as shown for H3K27me3 and upregulation of genes associated with neurogenesis, neuronal differentiation, and function. Further analysis demonstrates that several of these genes are downregulated in GBM, potentially through epigenetic silencing as indicated by the presence of H3K27me3 sites.

**Conclusions:**

SERBP1 is the first example of an RNA-binding protein functioning as a central regulator of cancer metabolism and indirect modulator of epigenetic regulation in GBM. By bridging these two processes, SERBP1 enhances glioma stem cell phenotypes and contributes to GBM poorly differentiated state.

## Background

Glioblastoma (GBM) is the most common type of brain tumor with 13,000 new cases each year in the USA alone. Unfortunately, relapse occurs in almost every case with an average survival of 14 months after diagnosis. The data generated by large cancer genomics consortia have become a critical tool to develop new therapeutic approaches against very aggressive tumors like GBM. In this respect, The Cancer Genome Atlas (TCGA) has produced an extensive transcriptomic map, identified prevalent chromosomal alterations, and defined important GBM driver mutations [1]. This knowledge has improved molecular classification, but thus far, therapeutic strategies based on these findings have not yielded a major breakthrough. Post-transcriptional processes such as splicing, poly adenylation, decay, and translation are often linked to tumorigenesis while many of their regulators have been shown to display tumor suppressive or oncogenic activity [2, 3]. Thus, studies on RNA-binding proteins (RBPs) that regulate these processes have the potential to expand our knowledge of GBM signaling and open up new avenues for therapy.

Over 1500 RBPs have been cataloged in the human genome [4]. Changes in their levels or functional activity lead to multiple alterations in RNA processing and/or expression that eventually contribute to acquisition of cancer-relevant phenotypes [3]. Despite their relevance and the fact that RBPs are largely altered in tumor tissue, the number of well-characterized RBPs in the context of tumorigenesis is still very small. RBPs are particularly relevant in the nervous system where splicing and translation regulate critical aspects of neurogenesis, neuronal function, and nervous system development [5]. Not surprisingly, RBPs are important oncogenic factors in medulloblastoma and gliomas [6].

We have identified SERBP1 (Serpine1 mRNA-binding protein 1) as a new oncogenic factor in GBM. SERBP1 is a member of the RG/RGG family of RNA-binding proteins, known for their involvement in neurological and neuromuscular diseases and cancer. SERBP1 regulates the expression of Serpine1 (PAI-1) [7], a member of the serine protease inhibitor [8]. SERBP1 may contribute to development of numerous tumor types. It has been identified as a target of the tumor suppressor miRNA miR-218 in hepato-cellular carcinoma (HCC) and linked to cell migration/invasion and epithelial-mesenchymal transition [9]. SERBP1 is markedly upregulated in prostate cancer and significantly associated with tissue metastasis and Gleason score [10]. It was also recently identified in a screening for driver genes mediating progression of androgen-independent prostate cancer (AIPC) using a xenotransplant mouse model [11]. In ovarian cancer, SERBP1 expression is higher in advanced disease, suggesting it plays a role in invasion and metastasis [12]. Finally, a Bayesian network study identified SERBP1 as part of the most influential gene signature in GBM development [13].

SERBP1 regulates mRNA translation [14–16], but known protein patterns suggest additional regulatory functions. Vig and vig2, SERBP1 Drosophila homologs, encode RNAi complex components that are involved in heterochromatin formation [17] and were recently identified in a CRISPR screening for histone gene regulators [18]. SERBP1 also was identified as a partner of Receptor for Activated C Kinase 1 (RACK1) in a two-hybrid screening [19] and interacts with arginine-methylated and stress granule-associated proteins like heterogeneous nuclear ribonucleoprotein A1 (hnRNPA1) and fragile X mental retardation protein (FMR1) [20]. SERBP1 also interacts with ribosomal proteins, congruent with its role in regulating translation [14].

In the present study, we investigated the role of SERBP1 in GBM development. High SERBP1 expression levels were linked to poor patient outcome and response to therapy. SERBP1 knockdown produced strong effects on cancer-related phenotypes and tumor growth. Genomic analysis indicated that SERBP1 is a critical player in cancer metabolism. An important consequence of this regulation is that SERBP1 modulates methionine production, affecting histone methylation and subsequently expression of neuronal differentiation genes. Overall, our results established SERBP1 as a novel oncogenic factor and a potential therapeutic target.

## Results

### SERBP1 is a novel prognostic marker in GBM

**Fig. 1:**
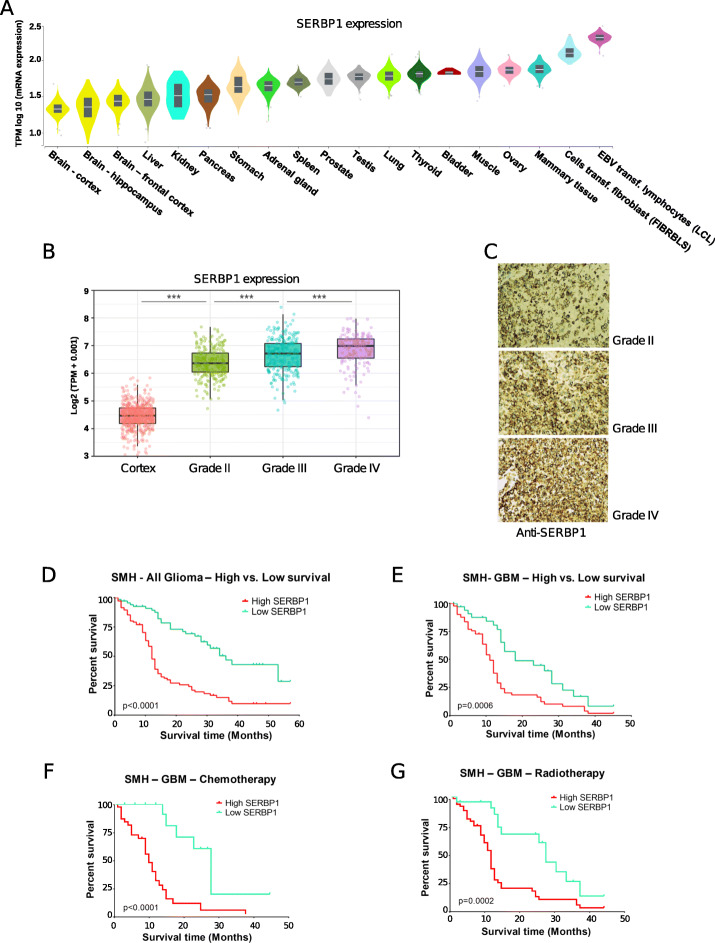
SERBP1 expression and impact on glioma survival and therapy. **a** SERBP1 mRNA expression in normal human tissue based on the GTEx database. **b** Comparative analysis of SERBP1 mRNA expression in normal brain/cortex (GTEx) and glioma samples (grades II, III, and IV) from the TCGA consortium. **c** Immunostaining of representative glioma samples (grades II, III, and IV) from the Shanghai Hospital cohort showing SERBP1 protein expression levels. **d** Kaplan–Meier curves indicate the survival of 177 glioma patients from the Shanghai Changzheng Hospital cohort displaying low and high SERBP1 levels. **e**–**g** Kaplan-Meier curves indicate the survival of 118 GBM patients from the Shanghai Changzheng Hospital cohort displaying low and high SERBP1 levels: E (all patients), F (54 patients who received TMZ), and G (83 patients who received radiation)

We evaluated the role of SERBP1 as an oncogenic factor in glioma/GBM and its potential impact on patient survival and response to therapy. In a multi-tissue transcriptomic analysis performed with the GTEx dataset [21], brain tissues show reduced SERBP1 mRNA expression while two transformed cell lines (LCL and FIBRBLS) showed the highest levels (Fig. 1a). Notably, SERBP1 shows higher expression in GBM (TCGA samples) compared to normal brain and LGG (low-grade glioma, grade II) (Fig. 1b, c, Additional File 2: Table S1). High SERBP1 expression was associated with poor survival in glioma patients in the TCGA and CGGA cohorts (Fig. S1A, Additional File 3: Table S2). Moreover, we observed that SERBP1 displays increased levels of expression in 25 tumor types when compared to normal tissue (Additional File 1: Fig. S1B) and data in the R2 database indicated that high SERBP1 expression is associated with poor prognosis in the cases of neuroblastoma, pancreatic adenocarcinoma, bladder urothelial carcinoma, cervical squamous cell carcinoma, and sarcomas (Additional File 1: Fig. S1C).

To corroborate and expand these results, we conducted a study using a glioma cohort from the Shanghai Changzheng Hospital. Samples were analyzed via immunostaining; SERBP1 was not detected in 21.5% of the samples, was mildly positive in 18.1%, was moderately positive in 37.3%, and was strongly positive in 23.2% of patients. We further examined whether SERBP1 expression was associated with glioma grades. SERBP1 was positive in 28.6% of those with grade I gliomas, 55.6% of those with grade II gliomas, 83.3% of those with grade III gliomas, and 89.0% of those with grade IV gliomas (*P* < 0.001). Moreover, 71.2% of patients with grade IV (GBM) had high positive staining for SERBP1, significantly more than grade I (7.1%), grade II (40.7%), and grade III (61.1%) gliomas (*P* < 0.001) (Fig. 1c, Additional File 4: Table S3). SERBP1 expression was closely correlated with WHO grade of glioma (low grade vs. high grade, chi-square test, *P* = 0.002) (Additional File 4: Table S3). Despite variable expression of SERBP1 in gliomas, GBM samples had noticeably higher expression of SERBP1 compared to LGG samples. The median survival was 13.12 ± 1.12 months (95% confidence intervals [CI] 10.91–15.33) for patients with high SERBP1 expression and 23.73 ± 1.76 (95% CI 20.23–27.23) months for patients with low SERBP1 expression (*P* < 0.0001) (Fig. 1d). GBM patients with low SERBP1 expression had markedly longer survival (17.41 ± 1.93 months, 95% CI 13.48–21.35) than those with high SERBP1 expression (10.52 ± 0.97 months, 95% CI 8.59–12.46) (*P* = 0.0006) (Fig. 1e).

We also examined whether survival time was influenced by postoperative adjuvant therapy (radiotherapy and chemotherapy). As shown in (Fig. 1f), among GBM patients who received chemotherapy (temozolomide), those with low SERBP1 levels had significantly longer survival (20.91 ± 2.52 months, 95% CI 15.67–26.15) than those with high SERBP1 levels (11.11 ± 1.22 months, 95% CI 8.68–13.55) (*P* = 0.0002). Similarly, patients with low SERBP1 levels gained more benefit from radiotherapy in terms of survival (18.81 ± 2.69 months, 95% CI 13.09–24.54) than those with high SERBP1 levels (9.24 ± 1.15 months, 95% CI 6.90–11.57) (*P* < 0.0001) (Fig. 1g). All patient data are listed in (Additional File 5: Table S4).

### SERBP1 influences cancer-relevant phenotypes

We conducted several assays to determine if high expression of SERBP1 is required to maintain cancer-relevant phenotypes. All experiments were performed with siRNA knockdown since attempts to produce SERBP1 knockout lines using the CRISPR-Cas9 approach were not successful, suggesting that at least the GBM lines we tested are unable to survive without SERBP1 function.

**Fig. 2:**
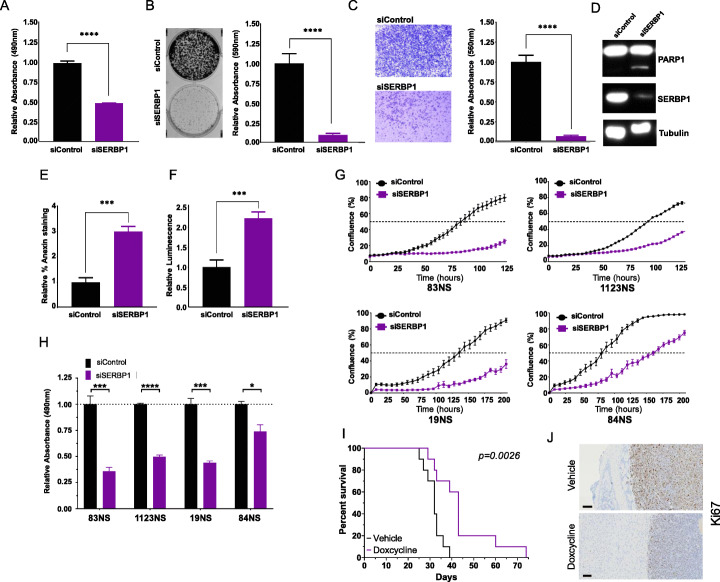
SERBP1 affects cancer-related phenotypes and tumor growth. **a** Knockdown of SERBP1 in U251 cells decreased viability (MTS assay). **b** SERBP1 silencing diminished clonogenic potential, as measured by colony formation assays. **c** The Boyden chamber assay was used to evaluate SERBP1 impact on invasion; values of crystal violet absorbance showed that SERBP1 knockdown decreased invasion potential. **d**–**f** SERBP1 silencing increased apoptosis as indicated by PARP1 cleavage (**d**), annexin staining (**e**), and caspase (**f**). **g** GSC proliferation across time was followed with the Incucyte automated system. Decrease in SERBP1 levels impaired cell proliferation. **i** Knockdown of SERBP1 in GSC lines decreased viability (MTS assays). Data were analyzed with Student’s *t* test and presented as the mean ± standard deviation. Bonferroni correction was used for multiple comparisons. *p ≤ 0.05; **p ≤ 0.01; ***p ≤ 0.001; ****p ≤ 0.0001. **i** Intracranial xenografts were established using the shSERBP1 3565 GSC cell line (10 mice per group). Experimental group received Dox to induce expression of shSERBP1. Kaplan-Meier curves indicate that SERBP1 knockdown decreased tumor growth and expanded survival. **j** Representative Ki67 staining from the tumor boundary for each group. Scale bar = 100 μm

SERBP1 knockdown caused a dramatic impact on U251 and U343 cell viability as detected via MTS assay (Fig. 2a, Additional File 1: Fig. S2A, S3A) We also conducted a cell colony formation assay and observed in both lines that a decrease in SERBP1 expression dramatically reduced GBM cell ability to form colonies (Fig. 2b, Additional File 1: Fig. S3B). Similarly, using the Boyden chamber assay, we determined that cell invasion was compromised when SERBP1 was silenced (Fig. 2c, Additional File 1: Fig. S3C). Next, we investigated if SERBP1 knockdown affects apoptosis. We examined PARP1 cleavage by Western blot as well as annexin-V staining using flow cytometry; in both scenarios, we observed an increase in product in cells transfected with siSERBP1 compared to control siRNA (Fig. 2d, e). Similarly, we observed a higher caspase 3/7 activity in SERBP1 knockdown cells compared to control, corroborating the role of SERBP1 in apoptosis (Fig. 2f). Finally, we evaluated the impact of SERBP1 on glioma stem cell (GSC) survival and proliferation. Geltrex was used to allow GSC cultures to grow as monolayers. Survival was evaluated using the MTS assay while cell proliferation was followed over time using the Incucyte system. SERBP1 knockdown decreased survival and the proliferation of both proneural and mesenchymal GSCs (Fig. 2g, h; Additional File 1: Fig. S2B).

Next, we evaluated the impact of SERBP1 on tumor growth using intracranial xenografts. We selected the highly aggressive GSC line 3565 [22] and prepared a stable line containing a tet-inducible SERBP1 shRNA via lentiviral infection. Evaluation of this line in vitro showed that 80% knockdown can be achieved upon treatment with doxycycline. Reduction in neurosphere formation and survival were observed upon treatment with doxycycline and results correlated with the amount of drug used (Additional File 1: Fig.S2C-F). Both control group and experimental groups (5 males and 5 females/group) were implanted with 3565 SERBP1 shRNA-tet cells. In the experimental group, cells were treated with doxycycline prior to implantation and mice were allowed to drink water containing doxycycline ad libitum. By reducing SERBP1 expression in the experimental group, we extended the median survival by 11 days versus controls group (*P* = 0.0026) (Fig. 2i). Tumors also showed very different morphology. As reflected by the immunostaining with anti-Ki67, there is a marked difference in the number of highly proliferating cells (Fig. 2j). SERBP1 expression profile, its impact on GBM patient survival, response to therapy, cancer phenotypes and tumor growth establish SERBP1 as a new oncogenic RBP in GBM.

### Characterization of SERBP1-binding motif and regulatory impact

**Fig. 3:**
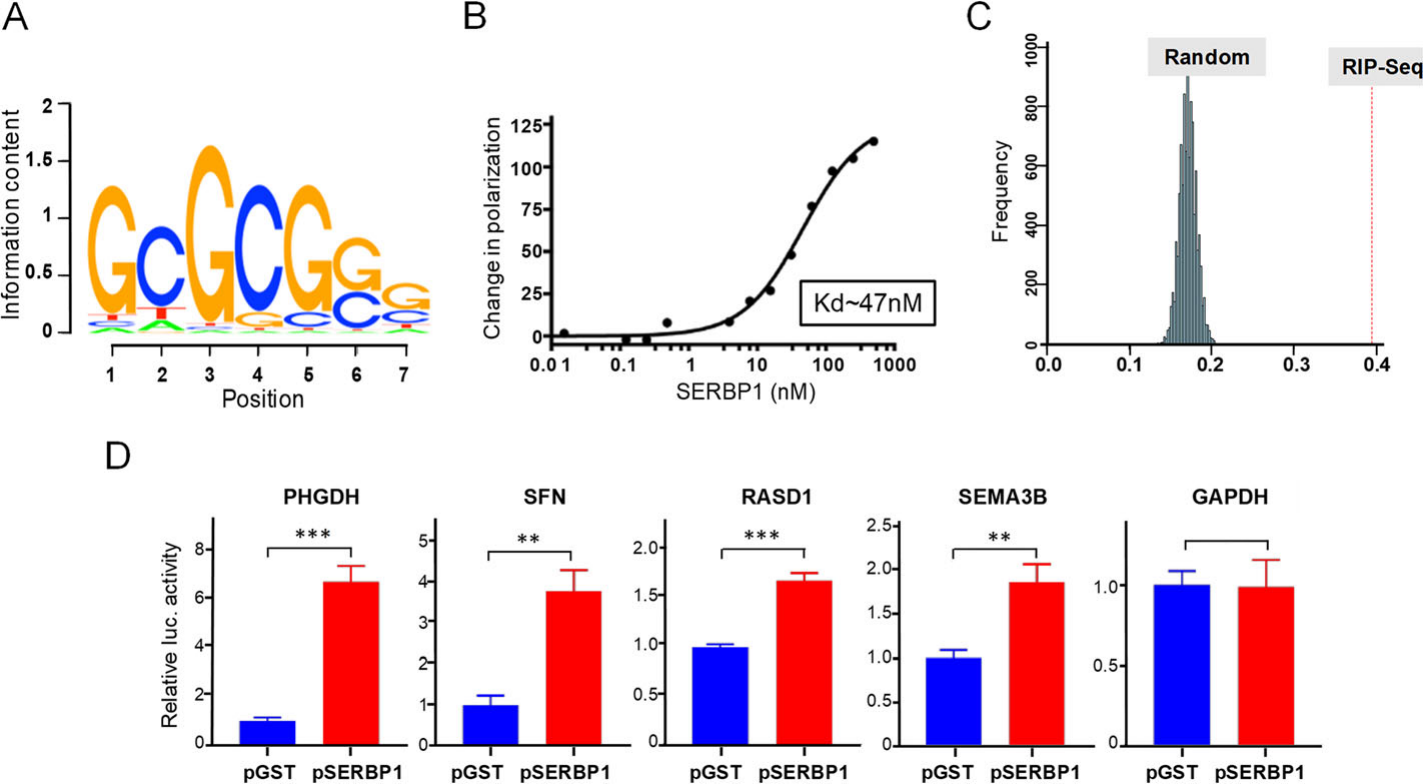
SERBP1 binding motif. **a** SERBP1 RNA binding motif obtained with RNACompete. **b** Fluorescence polarization assay shows SERBP1’s high affinity (K_D_ ~ 47 nM) to a 7-mer RNA oligonucleotide (5′- GCGCGGG - 3′). **c** ~ 40% of transcripts determined via RIP-Seq as preferentially associated with SERBP1 display the identified GC-rich motif in their 3′ UTR, a number much higher than expected by chance. **d** Results of luciferase assay showing that co-transfection of a SERBP1 expression vector increased the expression of reporter constructs containing the 3′ UTR of genes displaying putative SERBP1 binding motifs. GAPDH was used as a negative control

To define SERBP1 RNA-binding preferences, we used RNAcompete [23]. Full-length recombinant SERBP1 protein was incubated with a pool of ~ 240,000 designed (non-random) RNA oligos. RNAs selected by SERBP1 were identified via microarray hybridization and subsequent computational analysis identified a GC-rich RNA-binding motif for SERBP1 (Fig. 3a). We also expressed and purified SERBP1 as a N-terminus His_6_ fusion protein and used fluorescence polarization (FP) to quantitatively measure its binding affinity to an RNA 7-mer (5′- GCGCGGG - 3′), identified by RNAcompete. The measured equilibrium dissociation constant (K_D_) of SERBP1-RNA interaction (K_D_ ~ 47 nM) corroborates its strong affinity to the sequence identified by RNA Compete (Fig. 3b). To determine if SERBP1 binds to this motif in cells, we conducted a RIP-Seq experiment. We observed that ~ 40% of the mRNAs species determined to be bound by SERBP1 display the identified GC-rich motif in their 3′ UTR, a number that is much higher than expected by chance (Fig. 3c). Complete results of the RIP-Seq analysis are shown in (Additional File 6: Table S5). Finally, we selected genes with multiple “SERBP1-binding motifs” in their 3′ UTR that also were affected by SERBP1 knockdown (see RNA-Seq analysis below) to perform luciferase assays. Luc-reporters containing the 3′ UTR of these genes were co-transfected with a SERBP1 expression vector or control. In all cases, SERBP1 transgenic expression increased the expression of the luciferase reporter (Fig. 3d), indicating promotes mRNA stability and/or translation of these transcripts.

### SERBP1 is a regulator of “cancer metabolism”

**Fig. 4:**
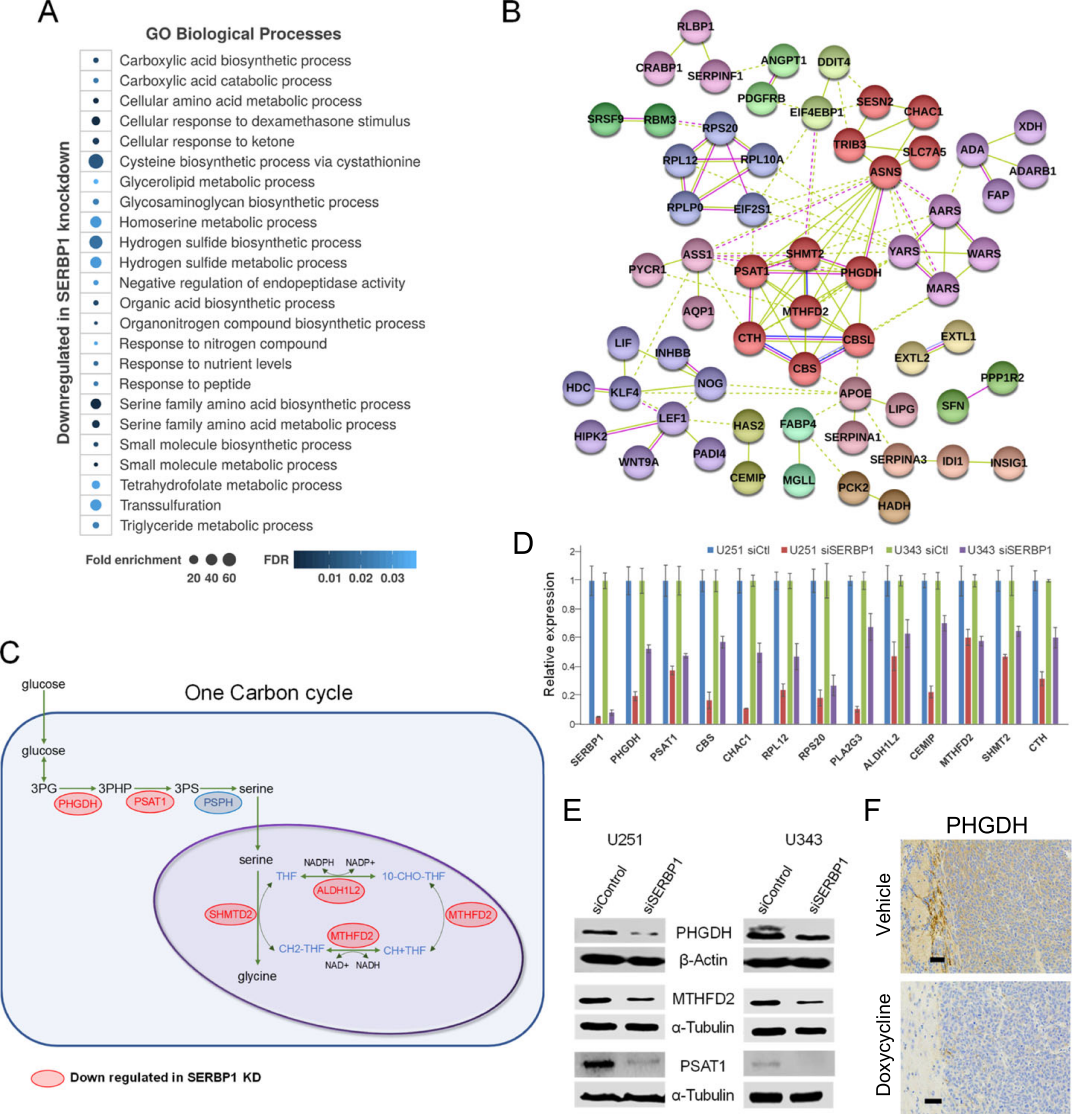
SERBP1 regulates metabolism. **a** Enriched Gene Ontology (GO) terms related to genes downregulated upon SERBP1 in U251 cells. Gene set was analyzed using Panther [24] and GO terms were compiled using Revigo [25]; most representative terms associated with metabolism are listed. **b** Network analysis of genes implicated in metabolism affected by SERBP1 knockdown. Network was built using String [26] considering interaction (experimental evidence), text mining, and co-occurrence. Different colors were used to indicate clusters. **c** Schematic representation of one-carbon cycle, showing genes affected by SERBP1 knockdown. **d**, **e** qRT-PCR and Western blot analysis in U251 and U343 cells corroborated the impact of SERBP1 on the expression of critical genes implicated in metabolism. **f** Representative PHGDH immunostaining of tumors from the xenograft study for each group. Scale bar = 60 μm

To understand how SERBP1 contributes to cancer-relevant phenotypes, we performed an RNA-Seq analysis in control vs. SERBP1 knockdown U251 cells. SERBP1 knockdown decreased expression of a large set of genes associated with metabolism and metabolism regulation as illustrated by the top enriched Gene Ontology terms (Fig. 4a, Additional File 7: Table S6). Network analysis showed that this set of genes associated with metabolism is highly interconnected (Fig. 4b). Similarly, RIP-Seq analysis determined that multiple transcripts (genes) bound by SERBP1 are implicated in metabolism (Additional File 7: Table S6). In particular, two interconnected metabolic routes, serine biosynthesis and one-carbon (1C) cycle, are highly affected by SERBP1 knockdown (Fig. 4c). Altered expression of genes linked to these pathways was validated by qRT-PCR and Western blot (Fig. 4d, e). Immunostaining of xenografts also showed that SERBP1 knockdown also produced a dramatic decrease in PHGDH expression in tumors (Fig. 4f).

One-carbon (1C) metabolism is a universal folate-dependent pathway that produces 1C units used for de novo purine and thymidylate synthesis, interconversion of several amino acids, production of universal methyl donors, and regeneration of redox cofactors, all of which benefit cancer survival [27]. Targeting of some 1C enzymes has already been explored as a therapeutic strategy [27–29]. Relevant 1C genes include PHGDH (3-phospho-glycerate dehydrogenase), SHMT2 (serine hydroxylmethyl-transferase 2), MTHFD2 (methylene tetrahydrofolate dehydrogenase 2), and PSAT1 (phosphoserine aminotransferase 1) (Fig. 4b). In cancer cells, 3-phosphoglycerate is used as part of a growth-promoting mechanism to synthesize serine and glycine and to generate NADPH. Half of glucose-derived carbon is used in serine biosynthesis and PHGDH functions as the limiting enzyme in this process [30]. PHGDH is overexpressed in glioma and affects invasion and angiogenesis [31]. SHMT2 is a critical enzyme in the 1C cycle [32] and controls a critical point in cancer metabolism—direction of serine/glycine conversion. Depletion of serine inhibits cancer cell proliferation and reduces purine levels, a similar effect is observed after SHMT2 knockdown [27]. MTHFD2 is a NAD+-dependent enzyme with dehydrogenase and cyclohydrolase activity implicated in mitochondrial 1C folate metabolism and it is one the most frequently metabolic enzymes overexpressed in tumors [33]. PSAT1 is a critical enzyme in the sub-pathway that synthesizes L-serine from 3-phospho-D-glycerate. High PSAT1 expression correlates with poor prognosis in many tumors including breast cancer, colorectal, nasopharyngeal, and esophageal carcinomas and is linked to drug resistance [34–37].

**Fig. 5:**
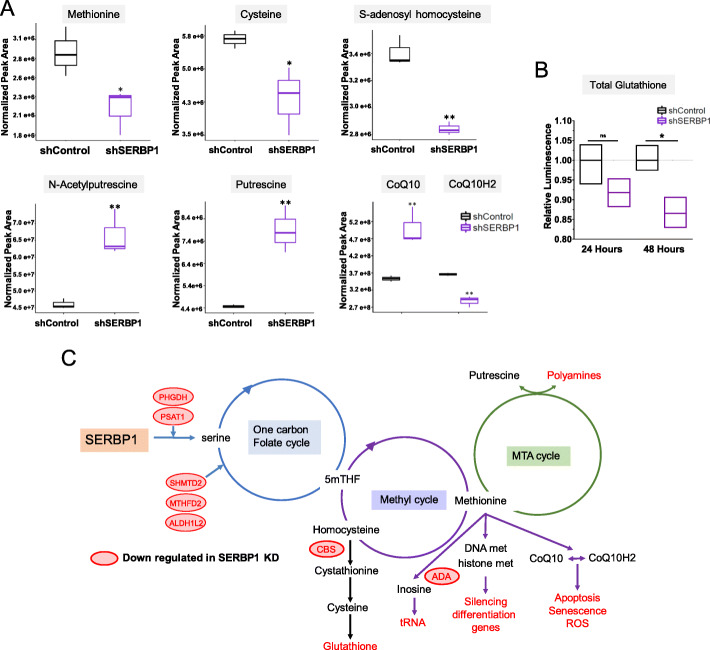
SERBP1 impact on one-carbon and methyl cycles and potential downstream effects. **a** Metabolic analysis shows that SERBP1 silencing affected the production of metabolites associated with one-carbon, methyl, and MTA cycles. **b** Intracellular glutathione levels following SERBP1 silencing. **c** Model for SERBP1 impact on metabolism and functional downstream effects

We conducted a metabolic study to corroborate the results of our RNA-Seq analysis. Since SERBP1 siRNA knockdown markedly affected viability and apoptosis, we opted to use a U251 stable line with a tet-inducible shRNA. Principal component analysis (PCA) performed on the metabolomics data shows a distinct separation between the control and the SERBP1 knockdown groups (Additional File 1: Fig. S4A-B). Volcano plot data shows a significant depletion of methyl cycle intermediates (S-adenosylhomocysteine and methionine) and accumulation of the 5-methylthioadenosine (MTA) cycle intermediates (putrescine and N-acetylputrescine) (Additional File 1: Fig. S4C). Metabolic analyses corroborated that SERPB1 modulates serine metabolism and interferes with the transfer of one-carbon unit (C1) from the folate cycle to the methyl cycle. This change could alter DNA/histone methylation, regeneration of redox cofactors, and nucleotides biosynthesis [27, 38, 39]. The effect is evident from the significant depletion of methionine and S-adenosylhomocysteine (SAH), the byproduct of methylation that recycles methionine through the contribution of a methyl group from 1C cycle intermediate methyl-tetrahydrofolate (methyl-THF) (Fig. 5a, c and Additional File 1: Fig. S4). High levels of methionine in the cell, as well as diets rich in methionine, have been associated with tumor progression [40, 41]. On the other hand, significant upregulation of the polyamine precursors, putrescine, and N-acetyl putrescine suggests the unavailability of methionine and its metabolite S-adenosylmethionine (SAM); SAM provides the amino-propyl group to putrescine for polyamine synthesis through the MTA cycle [42]. Furthermore, depletion of cysteine is linked to the downregulation of cystathionine-β-synthase (CBS) which catalyzes biosynthesis of cysteine from SAH, having homocysteine and cystathionine as intermediates [38].

A decrease in the total levels of glutathione was determined by a luminescent-based assay (Fig. 5b). Since cysteine is a precursor of glutathione (GSH), a redox-regulating metabolite, its depletion might affect the redox balance in SERPB1 knockdown cells (Fig. 5c). Similarly, significant depletion of coenzyme Q_10_ (CoQ_10_H_2_) or a high CoQ_10_/CoQ_10_H_2_ ratio in the knockdown group suggests dysregulation of the mitochondrial respiratory chain (mETC), inhibition of oxidative phosphorylation, and generation of reactive oxygen species (ROS) that ultimately trigger apoptosis [43]. CoQ10 is a lipophilic redox cofactor that functions as an electron carrier in the mETC and generates the proton gradient that drives ATP synthesis through oxidative phosphorylation [44] (Fig. 5a, c). In summary, the results of the metabolic analysis support the changes observed in the genomic study, establishing SERBP1 as a novel regulator of metabolic pathways. Full results of the metabolic analysis are shown in (Additional File 8: Table S7).

### SERBP1 impact on neuronal differentiation, “stemness,” and epigenetic regulation

**Fig. 6:**
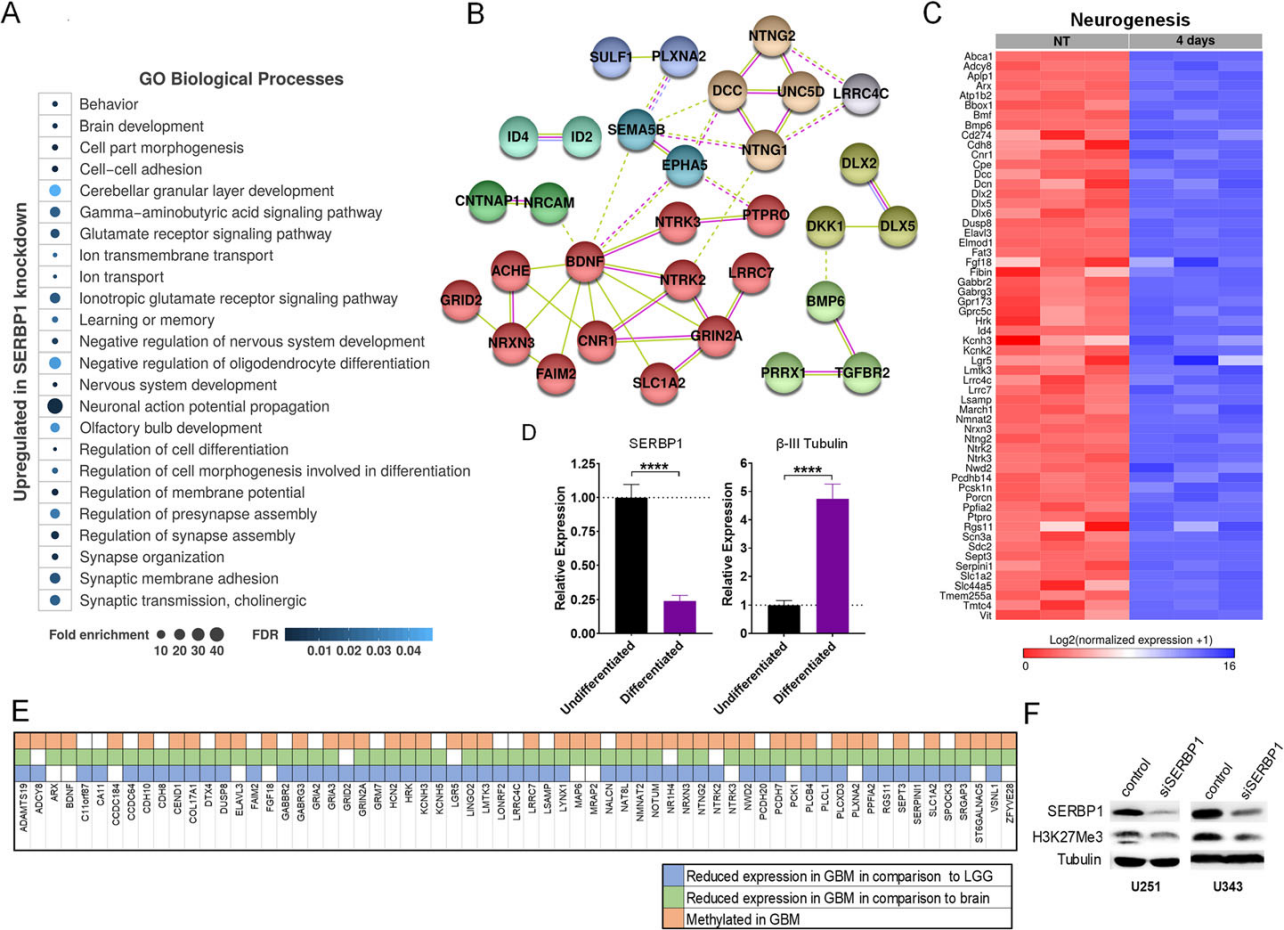
SERBP1 knockdown increased expression of genes linked to neurogenesis and nervous system development. **a** Enriched Gene Ontology terms related to genes upregulated upon SERBP1 knockdown in U251 cells. Gene set was analyzed using Panther [24] and GO terms were compiled using [25]. Most representative terms associated with nervous system development and function are listed. **b** Network analysis of genes implicated in neuronal differentiation affected by SERBP1 knockdown. The network was built using String [26] considering interaction (experimental evidence), text mining, and co-occurrence. Different colors were used to indicate clusters. **c** Heatmap shows that genes upregulated upon SERBP1 knockdown cells display increased expression during murine neurogenesis—0 day/stem vs. 4 days/differentiated cells. **d** qRT-PCR shows SERBP1 and β-III Tubulin expression in neuronal stem cells (NSCs) and differentiated cells. **e** Genes upregulated upon SERBP1 knockdown showing decreased expression in GBM in reference to LGG are labeled in blue, genes that show reduced expression in GBM in reference to normal brain (cortex) are labeled in green and genes that are methylated (H3K27me3) in GBM cells [45] are labeled in orange. **f** Western blot showing that SERBP1 knockdown leads to a decrease in H3K27me3 in GBM cells

Genes upregulated after SERBP1 knockdown are preferentially associated with nervous system development, neurogenesis, and synaptogenesis according to GO analysis (Fig. 6a, b; Additional File 1: Fig. S5, Additional File 7: Table S6). This data suggests that SERBP1 could function as a “repressor” of neuronal differentiation. In fact, according to our previous analysis [46], most of these genes show an increase in expression during neurogenesis (Fig. 6c). SERBP1 shows the opposite pattern; NSCs and GSCs display high levels of SERBP1 while decreased expression occurs when cells are induced to differentiate (Fig. 6d, Additional File 1: Fig. S6A-B). Next, we performed gene expression correlation analyses with R2 using TCGA GBM and brain samples. Genes showing strong negative correlation with SERBP1 are found in many GO-enriched categories previously identified in analyses of genes upregulated upon SERBP1 knockdown (e.g., synapse organization, nervous system development, and neurogenesis) (Additional File 9: Table S8, Additional File 10: Table S9). Overall, these results suggest that SERBP1 represses neuronal differentiation. We then evaluated whether increased SERBP1 expression can disrupt neuronal differentiation using the neuroblastoma BE-(2)-C cell line. Cells were infected with either SERBP1 expressing or control lentivirus and treated with or without retinoic acid (RA) to induce differentiation. After 4 days, we used an Incucyte system to measure neurite outgrowth as an indicator of differentiation. RA treatment effectively induced neurite formation but SERBP1 overexpression diminished the effect (Additional File 1: Fig. S6C-E).

The effect of SERBP1 on methionine production could ultimately influence histone and/or DNA methylation. Therefore, SERBP1 could function as an epigenetic modulator, indirectly repressing the expression of genes implicated in neuronal differentiation. Gene set enrichment analysis (GSEA) identified strong similarities between the list of upregulated genes in SERBP1 knockdown cells and binding profiles of H3K27me3 and EZH2 and SUZ12, two members of PRC2 which trimethylate histone H3 on lysine 27 (Additional File 11: Table S10). Analysis of H3K27me3 profiles in GBM cells [45] confirmed that several genes “repressed” by SERBP1 show H3K27me3 sites (Fig. 6e, Additional File 12: Table S11). Consistent with these results, levels of expression for most of these genes are decreased in GBM samples in comparison to brain (cortex) and LGG (Fig. 6e, Additional File 13: Table S12. Corroborating the association between SERBP1 and H3K27me3, we determined by Western analysis that SERBP1 knockdown in GBM cells reduced H3K27me3 levels (Fig. 6f).

We then tested if a reduction in SERBP1 levels could increase GBM cell sensitivity to PRC2 inhibition. EED226 is a potent and selective PRC2 inhibitor that directly binds to the H3K27me3 binding pocket of EED. Control and SERBP1 knockdown cells were treated with 20 or 40 μM of EED226 and cell proliferation was followed over time with an Incucyte. Since the impact of SERBP1 on cell proliferation is very strong, we decided to use a partial SERBP1 knockdown (40–50% reduction in proliferation) to appreciate the effect of combined treatment. While control cells showed almost no response to EED226 treatment, SERBP1 knockdown cells showed decreased proliferation in response to treatment (Additional File 1: Fig. S7A,S7C). To better illustrate the differences between siRNA control and SERBP1 knockdown cells, we show proliferation at the terminal time point, comparing EED226-treated cells to its respective untreated control (Additional File 1: Fig. S7B, S7D).

In line with these findings, we observed that SERBP1 knockdown decreased expression of several genes associated with PI3K/AKT signaling (Additional File 1: Fig. S8A, Additional File 7: Table S6) which has been shown to modulate the cancer epigenome through methylation [47]. Corroborating the connection between SERBP1 and the PI3K/AKT pathway, knockdown of SERBP1 reduced levels of AKT and p-AKT in U251 and U343 cells as observed by Western blot (Additional File 1: Fig. S8B). Finally, using the glioma cohort from the Shanghai Changzheng, we determined that AKT1 and SERBP1 display good expression correlation based on immunostaining (Additional File 1: Fig. S8C).

Finally, we examined whether increased expression of SERBP1 could contribute to stem cell features. We generated, via lentiviral infection, a U343 line that overexpresses SERBP1 (SERBP1 OE) (Additional File 1: Fig. S2G-H). Control (empty vector) and SERBP1 OE cells were grown as neuro-spheroids in stem cell media. SERBP1 OE cells were more efficient in forming spheres than controls and the difference increased in higher passages (Additional File 1: Fig. S9A-B). SERBP1 OE cells also showed increased expression of stem cell markers by qRT-PCR (Additional File 1: Fig. S9C). Increased metabolism and radio-resistance are characteristics of “cancer stem cells” [48]. We observed that SERBP1 OE cells also had increased mitochondrial respiration and ATP production compared to controls (Additional File 1: Fig. S9D-E). Consistent with the finding that that high SERBP1 expression influences response to radiation in GBM patients (Fig. 1G), SERBP1 OE cells were more resistant to radiation than controls as shown in a colony formation assay (Additional File 1: Fig. S9F).

Overall, our results indicate that SERBP1 contributes to GBM poorly differentiated state and glioma stem cell phenotypes by repressing genes implicated in neuronal differentiation and neuronal function. SERBP1 could influence epigenetic regulation by controlling methionine production via the one-carbon and methyl cycles and the AKT pathway.

## Discussion

### SERBP1 as a new regulator of cancer metabolism

Metabolic reprogramming is a hallmark of cancer [49]. Cancer cells capture and use nutrients more efficiently and repurpose metabolic pathways, creating alternatives for energy and biomass production. Clinical and preclinical data strongly suggest that targeting metabolism is a realistic strategy to treat aggressive tumors like GBM [50]. SERBP1 is perhaps the first example of an RNA-binding protein functioning as a central regulator of metabolic pathways, coordinating expression of related enzymes and associated factors implicated in serine biosynthesis, and MTA cycles. Consistent with this claim, the depletion of key methyl and MTA cycle metabolites (e.g., methionine, SAH, and cysteine) in SERBP1 knockdown cells indicates that SERBP1 controls a variety of processes including methylation of proteins, DNA, RNA, and lipids as well as biosynthesis of nucleotides and polyamines [39]. SERBP1 contributes to glycolysis and TCA cycles through the regulation of methionine, which helps generate redox cofactors necessary to keep dynamic processes such as glycolysis and TCA cycles functioning [51]. In fact, SERBP1 could have a much broader impact on metabolism, since our RIP-Seq analysis indicated that SERBP1 binds preferentially to transcripts of genes implicated in other metabolic pathways, such as mTOR. All these pathways are critical to cancer cells by regulating their ability to differentiate and quench oxidative stress. Regulation of these mechanisms by SERBP1 may elucidate the pathways by which aggressive cancers thrive.

In the set of genes upregulated in SERBP1 knockdown cells, we also identified a list of genes linked to metabolism. Several genes showed reduced expression in GBM versus normal brain and are likely to be epigenetically regulated by PRC2. Among the ones known to have tumor suppressive functions, we have focused on carboxypeptidase C (CPE), phosphoenolpyruvate carboxykinase 1 (PCK1), and phospholipase C-like 1 (PLCL1).

CPE is an enzyme that catalyzes the release of C-terminal arginine or lysine residues from polypeptides and is involved in the biosynthesis of neuropeptides and hormones [52]. In a glioma cohort, loss of CPE predominantly occurred in GBMs and was associated with a worse prognosis. Overexpression of CPE diminished glioma cell migration [53]. An increase in secreted CPE (sCPE) affected mRNAs levels of genes connected to the motility-associated networks, such as FAK, PAK, Cdc42, integrin, STAT3, and TGF-β [54]. Moreover, sCPE enhanced glucose flux into the tricarboxylic acid cycle at the expense of lactate production; this change reduced aerobic glycolysis, possibly contributing to a less invasive behavior of tumor cells [55].

PCK1 is a key enzyme in the gluconeogenesis pathway. PCK1 is downregulated in hepatocellular carcinoma (HCC) and clear cell renal cell carcinoma and its reduced expression predicts poor prognosis [56, 57]. Overexpression of PCK1 decreased viability, induced apoptosis, and inhibited migration of HCC cell lines [58]. The effect was associated with the suppressed glycolysis and induction of gluconeogenesis pathways [59]. Overexpression of PCK1 in glucose-starved HCC cells induced TCA cataplerosis, leading to energy crisis and oxidative stress [58]. PCK1 overexpression has been used to reprogram tumor-reactive T cells and enhance anti-tumor T cell responses. PCK1-overexpressing T cells restricted tumor growth and increased survival of melanoma-bearing mice [60].

PLCL1 is downregulated in clear cell renal carcinoma (ccRCC) and predicted a poor prognosis. Restoration of PLCL1 expression in ccRCC cells repressed tumor progression, reduced abnormal lipid accumulation, and caused tumor cell “slimming” through UCP1-mediated lipid browning, which consumes lipids without producing ATP energy [61].

In agreement with SERBP1’s positive effect on expression of genes associated with 1C and methyl cycles, SERBP1 knockdown produced marked changes in the levels of several metabolites including methionine. Disruption in methionine production can affect gene expression by interfering with DNA, RNA, and histone methylation [62]. Methyl groups necessary to fuel methionine regeneration are supplied by methyl donors from the methyl cycle [39], many of which were downregulated in our metabolomic analysis of SERBP1. In GBM, alterations in epigenetic regulation are a critical component of tumor development [63]. For instance, increased expression of specific histone methyltransferases is often observed in gliomas and causes aberrant methylation of lysine residues such as H3K4 and H3K27 [63]. Inhibitors against methyltransferases, in particular PRC2 inhibitors, are being tested in clinical trials of many different tumor types [64]. Although the full impact of SERBP1 on epigenetic regulation remains to be assessed, our data show an important association between SERBP1 and H3K27me3 levels and provides a logical explanation for increased expression of genes implicated in neuronal differentiation and synaptogenesis in SERBP1 knockdown cells.

### SERBP1’s potential impact on neuronal differentiation, synaptogenesis, and nervous system development

SERBP1 function in the nervous system is virtually unknown. Its expression is lower in the brain compared to other tissues. Genes implicated in neurogenesis, synaptogenesis, nervous system development, and function show strong negative expression correlation with SERBP1 both in brain and GBM samples and increased expression in SERBP1 knockdown cells. In agreement, we observed that SERBP1 expression decreases as NSC and GSC differentiate and that SERBP1 enhanced “stemness.” Altogether, this data suggests that SERBP1 might be critical for neuronal function and its aberrant expression could be linked to neurological disorders. Consistent with this idea, SERBP1 and proteins implicated in synaptic structure and morphogenesis were increased in the brains of a murine model of fragile X mental retardation [20]. Altered synaptic structure and function are major characteristics of fragile X syndrome [65].

Among genes affected by SERBP1 with critical roles in nervous system development and function, we focus on DCN, NTNG2, and SLC1A2. Decorin (DCN) was the top upregulated gene in SERBP1 knockdown analysis. DCN is a member of the small leucine-rich proteoglycan (SLRP) family that has been recently identified as a novel neurogenic factor in the central nervous system and as a critical component of the integrin-Wnt7a-Decorin pathway that promotes proliferation and differentiation of neuroepithelial cells [66]. It has been implicated in tumor development and metastasis and its decreased expression has been observed in various tumor types including gliomas. Importantly, when used as a therapeutic molecule in cancer models, DCN was able to block tumor progression and metastases [67]. In the case of GBM, DCN expression inhibited cell growth in vitro, promoted cell differentiation, suppressed tumor growth, and increased survival [68].

NTNG2, a gene that encodes Netrin-G2, is required for proper axonal guidance during early brain development and synaptic transmission [69]. Several mutations in NTNG2 have been described and they cause global developmental delay, hypotonia, secondary microcephaly, Rett-like phenotype, and autistic features [70–72]. Knockdown of murine Ntng2 caused severe impairments of neuronal morphology and cortical migration [71] while knockout of Ntng2 in a cellular model resulted in short neurites [72].

SLC1A2 is a member of the solute transporter family that clears the excitatory neurotransmitter glutamate from the extracellular space at synapses, allowing proper synaptic activation and preventing neuronal damage. Dysfunction or reduced expression of SLC1A2 has been linked to neurodegenerative diseases [73]. In gliomas, glutamate excitotoxicity induces neurodegeneration and necrosis. In this respect, inhibition of SLC1A2 in rats reduced glutamate uptake by glial cells and caused neuronal cell death [74].

### Targeting RNA-binding proteins in cancer therapy

RNA-binding proteins modulate gene expression from RNA processing to translation. Studies using TCGA data showed many mutations in RBPs and alterations in their expression levels across tumor types [2, 3]. RBPs can be targeted by small-molecule inhibitors as in the case of HuR and Musashi1 [75, 76] or by RNA aptamers or modified RNA oligos bearing the consensus binding motif of the RBP to be targeted. Targeting RBPs provides an opportunity to disrupt multiple cancer-relevant pathways at once. In the case of SERBP1, its blockage could hit cancer metabolism and epigenetic regulation.

## Conclusions

Our study defined SERBP1 as a novel oncogenic factor in GBM and as an indicator of prognosis and response to therapy. SERBP1’s role in regulating a gene network implicated in metabolic pathways relevant to cancer cells is likely to be a key contributor to its broad impact on cancer phenotypes and tumor growth. SERBP1 emerges as the first RBP to function as a critical modulator of metabolic pathways.

An important downstream effect of SERBP1 function is its impact on epigenetic regulation. SERBP1 ultimately impacts methionine production. Methionine levels influence DNA, RNA, and histone methylation. In particular, we observed that SERBP1 knockdown in GBM cells decreased methionine production causing a subsequent reduction in H3K27me3 levels and upregulation of genes associated with neurogenesis, synaptogenesis, neuronal differentiation, and function. We showed next that increased expression of SERBP1 prevents neuronal differentiation, enhances stem cell phenotypes, and resistance to radiation while SERBP1 knockdown increased sensitivity to epigenetic inhibitors. Overall, our results indicate that besides its role in GBM development, SERBP1 might be implicated in brain function and neurological disorders. Due to its broad impact on cancer-relevant processes and pathways, SERBP1 emerges as an important oncogenic factor and potential therapeutic target.

## Methods

### SERBP1 expression analysis in tumors and normal tissue

Count reads of glioma (grades II–IV) samples were first obtained from the TCGA data repository (https://portal.gdc.cancer.gov/) [1]. Raw sequencing reads were then extracted using SAMtools collate and fastq [77] and subsequently processed using Kallisto [78] with an index of 31 k-mers and GENCODE as a reference to the human transcriptome. Transcript abundance estimates were then collapsed into gene-level normalized read counts (TPM) using the R package tximport [79]. In addition, normalized read counts (TPM) data from healthy (frontal) cortex samples were directly obtained from the Genotype-Tissue Expression (release V8; https://www.gtexportal.org/home/) [21]. To process SERBP1 expression data in glioma and healthy cortex samples, TPM data was first log-transformed using R. Then, a box plot was built also using R and further edited in Inkscape (https://inkscape.org/).

Analyses of SERBP1 expression in normal tissues were conducted using the GTEx platform [21]. Normal (GTEx samples) and tumor (TCGA samples) tissues were compared using resources in Gepia [80]. We performed expression correlation analyses to identify genes with a strong positive or negative correlation with SERBP1 in the TCGA glioblastoma set (RNA-Seq samples) using R2 (https://hgserver1.amc.nl/cgi-bin/r2/main.cgi); *R* ≥ 0.3 or *R* ≤ − 0.3, *P* value 0.01 and correlation multiple testing: false discovery rate (FDR).

To identify SERBP1-regulated genes with changes in expression during neurogenesis, we used our previously published dataset in which we followed murine neurogenesis in vitro [46]. We selected genes with increased expression after SERBP1 knockdown and compared their expression levels in non-treated neural stem cells (NSCs) vs. differentiated cells (4 days after being transferred to differentiation media). Sequencing reads were processed using Kallisto [78] with an index of 31 k-mers and GENCODE (https://www.gencodegenes.org/; vM18) as the reference to the mouse transcriptome [81]. Transcript abundance estimates were next collapsed into gene-level counts using the R package tximport [79]. Differential expression analyses were performed between differentiated and undifferentiated NSCs using DESeq2 [82], and genes with an absolute log2FoldChange ≥ 1 and Benjamini-Hochberg (FDR) adjusted *P* value < 0.05 were considered differentially expressed. Next, expression levels of genes upregulated after SERBP1 knockdown were compared to those of genes differentially expressed between non-treated NSCs and differentiated cells. Finally, we built a heatmap for normalized expression levels of genes upregulated both upon SERBP1 knockdown and in differentiated NSCs, using the R package gplots (https://cran.r-project.org/package=gplots).

### SERBP1 expression in neurogenesis

VZ-SVZs were dissected under a stereoscope from 2-month-old Swiss-Webster mice (Charles River Laboratories) and dissociated to single cell suspension with 0.25% papain (Worthington) and 12 μg/ml DNase (Sigma) in DMEM at 37 °C for 45 min. Cells were washed 2× in DMEM by centrifugation and plated in a 6 well plate (Corning Inc.) in presence of N5 medium (DMEM/F12/N-2), 5% fetal bovine serum (FBS, Life Technologies), 20 ng/ml epidermal growth factor (EGF, Life Technologies), 20 ng/ml basic fibroblast growth factor (bFGF, Peprotech), and 35 μg/ml bovine pituitary extract (BPE, Life Technologies). Media was changed every 2 days. Differentiation of NSCs was then induced by removing EGF, FGF, and FBS from the media. Cells were collected at time 0 and 4 days for q-RT-PCR analysis of SERBP1 expression as described below. All animal care and experimental procedures were approved by the UTHSCSA Institutional Animal Care and Use Committee (Protocol #13091X).

### RNA extraction, RT-qPCR, and RNA-Seq

Total RNA from transfected cells was extracted using the TRIzol reagent (Invitrogen, Carlsbad, CA) according to the manufacturer’s instructions. Reverse transcription was performed using High-Capacity cDNA reverse transcription kit (Applied Biosystems) with random priming. Quantitative PCR was performed using TaqMan Universal PCR Master Mix (Applied Biosystems) or PowerUp SyBR Green Master Mix (Thermo Fisher) and reactions were performed on ViiA™ 7 Real-Time PCR System (Applied Biosystems). Data were acquired using ViiA 7 RUO software (Applied Biosystems) and analyzed using the 2−ΔΔCT method with GAPDH as an endogenous control. Probes and primers used in qRT-PCR are listed in (Additional File 14: Table S14).

Libraries used in RNA sequencing were prepared using TruSeq RNA Library Preparation Kit (Illumina), following the manufacturer’s instructions, and sequenced in a HiSeq-2000 machine in the UTHSCSA Genomic Facility.

### RIP-Seq

RIP-Seq experiments were conducted as we described previously [83]. A vector containing the Streptavidin-Binding Peptide (SBP)-Tag was prepared in pEF1 (Thermofisher) and SERBP1 or GST ORFs were cloned in frame with the tag. 293T cells were transfected with plasmids expressing SBP-SERBP1 or SBP-GST using CaCl_2_. Fresh media was added 12 h later, and cells were harvested 48 h later. Cell pellets were washed twice in cold PBS, frozen in dry ice, and stored at − 80 °C. To prepare the cell lysates, we thawed the tubes on ice and resuspended the cells in 2 volumes of polysomal lysis buffer (KCl 100 mM, EDTA 25 mM, MgCl2 5 mM, HEPES pH 7.0 10 mM, NP-40 0.5%, and glycerol 10%). Lysates were incubated on ice for 30 min and then sonicated 4 × 20 s at 20% amplitude with a 2-min interval. Cell lysates were then transferred to 1.5 ml tubes and later centrifuged at 15,000 RPM. Supernatant was collected and used later in RIP analysis.

Two hundred microliters of packed streptavidin beads (GE Healthcare Life Sciences) were washed five times in NT2 buffer and subsequently blocked in NT2 buffer + 5%BSA for 30 min at 4 °C. Beads were finally washed three times in NT2 buffer (Tris pH 7.4 50 mM, NaCl 150 mM, MgCl_2_ 1 mM, and NP-40 0.05%) and then combined with cell extract diluted in 5 volumes of NT2 buffer containing 25 mM EDTA, DTT, VRC, and RNase inhibitor. The solution was rotated at room temperature for 3 h and then centrifugated at 2000 RPM in a tabletop centrifuge for 5 min at 4 °C. The supernatant was discarded and beads were washed five times with 1 ml of NT2 buffer. To elute the RNA, samples were resuspended in elution buffer (Tris pH 7.4 50 mM, NaCl 250 mM, NP-40 0.5%, deoxicholate 0.1%, 10 mM biotin), mixed, and incubated for 30 min at 37 °C in a thermo-shaker at 1500 RPM. Beads were discarded after centrifugation, RNA was purified using the RNasey MinElute Cleanup kit (Qiagen) following the manufacturer’s instructions, and RNA was finally eluted in 20 μl RNase-free water. RNA samples were analyzed by RNA-Seq.

### RNASeq and RIP-Seq data analyses

RNA-Seq and RIP-Seq sequencing reads were processed using Kallisto [78] with an index of 31 k-mers and GENCODE (https://www.gencodegenes.org/; v28) as the reference to the human transcriptome [81]. Transcript abundance estimates were then collapsed into gene-level counts using the R package tximport [79]. Analyses of differential gene expression for both RNA-Seq and RIP-Seq datasets were performed using DESeq2 [82]. In the RNA-Seq analysis, we compared siRNA SERBP1 versus control samples and defined differentially expressed genes as those with an absolute log2FoldChange ≥ 1 and Benjamini-Hochberg (FDR) adjusted *P* value < 0.05. In RIP-Seq analysis, we compared SBP-SERBP1 versus SBP-GST samples and used log2FoldChange ≥ 0.5 and FDR adjusted *P* value < 0.05 to identify transcripts preferentially associated with SERBP1.

### Gene ontology (GO) and pathway enrichment analysis

To classify functions of differentially enriched genes, we performed GO and pathway enrichment using Reactome [84] and Panther [24]. For both analyses, we considered terms to be significant if *P* values were < 0.05 and fold enrichment was > 2.0 (adjusted for false discovery rates). Further, we used REVIGO [25] to reduce redundancy of the enriched GO terms and visualize the semantic clustering of the identified top-scoring terms. We used STRING database (v11) [26] to construct protein-protein interaction and determined associations among genes in a given dataset. The interactions were based on experimental evidence procured from high-throughput experiments, text-mining, and co-occurrence.

### Expression analysis of genes upregulated upon SERBP1 knockdown in GBM, LGG, and healthy cortex

To compare gene expression levels between samples ofd primary glioblastoma (GBM), low-grade glioma (LGG, grade II), and healthy (frontal) cortex, we first obtained aligned sequencing reads of 154 GBM and 248 LGG samples from the Cancer Genome Atlas - TCGA (https://cancergenome.nih.gov/). Raw sequencing reads were extracted using the SAMtools collate and fastq [77] and subsequently processed using Kallisto [78] with an index of 31 k-mers and GENCODE as reference to the human transcriptome. Transcript abundance estimates were then collapsed into gene-level counts using the R package tximport [79]. In addition, gene read counts of 464 samples from healthy (frontal) cortex were directly obtained from the Genotype-Tissue Expression dataset (release V8; https://gtexportal.org/). Differential expression analyses of GBM versus LGG and GBM versus healthy (frontal) cortex were performed using DESeq2 [82]. Genes with an absolute log2FoldChange ≥ 1 and Benjamini-Hochberg (FDR) adjusted *P* value < 0.05 were selected as differentially expressed. Finally, expression levels of genes upregulated upon SERBP1 knockdown were compared with those of genes downregulated in GBM compared to LGG and healthy cortex.

### Gene set enrichment analysis (GSEA) and methylation analysis

ENRICHR [85] was used to conduct GSEA and identify factors that potentially affect the expression of genes upregulated upon SERBP1 knockdown. The EZH2, SUZ12, and H3K27me3 ChIP-Seq datasets showing the greatest number matches were selected for further analysis.

To evaluate whether genes upregulated upon SERBP1 knockdown are methylated in GBM, we obtained Chip-Seq peak calls for H3K27me3 in 9 GBM samples from a previous study [45]. We also obtained transcriptional start sites (TSS) information for the human reference genome (GRCh38/hg38) from the Functional Annotation of the Mammalian Genome - FANTOM5 project (FANTOM Consortium and the RIKEN PMI and CLST) [86]; regions within − 3Kb to + 3Kb relative to each TSS were considered as putative promoter regions. Coverage of relevant H3K27me3 peaks (FDR adjusted *P* value < 0.05) was then calculated on each putative promoter using bedtools [87]. Only peaks observed in at least three samples were considered as indicative of methylation.

### Patient data collection, survival analysis, and therapy response

For immunohistochemical analyses, formalin-fixed, paraffin-embedded tissue sections, 3 μm thick, were deparaffinated in xylol and rehydrated in gradient ethanol. Antigen retrieval was performed by microwaving the tissue sections for 20 min in 1 mM EDTA buffer (pH 8.0). Endogenous peroxidase activity was eliminated by adding 0.3% hydrogen peroxide in methanol for 30 min. Slides were incubated in non-immune serum for 30 min followed by incubation with anti-SERBP1 primary polyclonal antibody (ab28481) or anti-AKT1 primary polyclonal antibody (ab18206) (Abcam Inc., Cambridge, UK) overnight at 4 °C. After washing in Tris-buffered saline with Tween-20, sections were incubated with biotin-conjugated secondary antibody for 20 min at room temperature, followed by 20 min incubation with peroxidase-conjugated biotin-streptavidin complex (Santa Cruz Biotechnology Inc., Santa Cruz, CA, USA). Tissue sections were then visualized by staining with 3, 3′-diaminobenzidin and counterstained with hematoxylin.

To evaluate immuno-reactivity, slides were examined in representative visual fields (× 400 magnification) to identify positively stained tumor cells. Intensity of positive staining was scored based on a scale of 0 to 3 (0, no detectable immunostaining; 1 light-brown color; 2, medium-brown color; 3, dark-brown color). The percentage of positivity in staining cells was also scored depending on the following cut points (0, no staining; 1, positive staining in < 25% of the tumor cells; 2, positive staining in 25–75% of the tumor cells; and 3, positive staining in > 75% of the tumor cells). Immuno-reactivity of cells was examined and calculated in five high-powered fields. The two scores were multiplied, and the results were referred to the expression score of the sample. All discrepancies in scoring were reviewed to reach a consensus. The total score representing the ratio of positive cells per specimen plus the intensity was defined as strong (+++ for total score ≥ 6), moderate (++ for total score = 4–5), weak (+ for total score = 1–3), or null (− for total score = 0). Results were further recorded as low or negative expression (+ and −) and high expression (++ and +++).

### Survival analysis in CGGA and TCGA glioma cohorts and other tumor types

To examine if SERBP1 expression levels are associated with survival among glioma patients (TCGA and CGGA consortia), we used the Gliovis platform [88]; a cut off of high vs. low expression was used to separate the patient groups.

Patient survival data were obtained from the R2 genomics analysis and visualization platform (https://hgserver1.amc.nl/cgi-bin/r2/main.cgi). We searched datasets to identify tumor types in which SERBP1 high expression clearly correlates with poor prognosis. Default parameters were used, Kaplan Scan (KaplanScan) established optimum survival cut-off based on statistical testing. Kaplan-Meier plots were generated based on the most optimal mRNA cut-off expression level to discriminate between cohorts with good or poor prognosis.

### Glioblastoma cell culture and transfections

Glioblastoma U251 and U343 cell lines were obtained from the University of Uppsala (Sweden) and cultured in Dulbecco’s modified Eagle’s medium (DMEM) supplemented with 10% fetal serum, 100 U/ml penicillin, and 100 μg/ml streptomycin. Cells were maintained at 37 °C in a 5% CO_2_ atmosphere. Cell lines were authenticated using short tandem repeat profiling.

Cells were reverse transfected with 25 nM of control or SERBP1 SMARTpool siRNA (Dharmacon) using Lipofectamine RNAiMax (Invitrogen, Carlsbad, CA) and harvested 72 h later for the assays described below. SERBP1 knockdown levels were consistent and exceeded 90% compared to the control siRNA transfection as measured by RT-qPCR and/or Western blots.

### Preparation of stable lines

The puromycin resistance gene was PCR amplified and cloned into pUltra (Addgene: #24129) XbaI and BamHI sites using Gibson Assembly (NEB) to generate the bicistronic eGFP-P2A-PuroR (pUltra control) vector. Subsequently, a plasmid containing the SERBP1 open-reading frame was obtained from the DNASU Plasmid Depository (HsCD00295647); the insert was PCR amplified and then cloned into the NheI/BclI sites to generate the multicistronic eGFP-P2A-PuroR-T2A-SERBP1 (pUltra-SERBP1) lentiviral vector. Vectors were propagated in Stbl3 cells (Thermofisher). Lentiviruses were prepared and titered as described previously [89]. U343 cells were infected with either pUltra or pUltra-SERBP1 virus at a MOI of 10. Forty-eight hours after transduction, cells were switched to puromycin-containing media (8 μg/ml) for 1 week, before being FACS based on high eGFP expression.

Tetracycline inducible shRNA lentiviral particles: SMARTvector Inducible Human SERBP1 hEF1a-TurboGFP shRNA and SMARTvector Inducible Non-targeting Control hEF1a/TurboGFP were obtained from Dharmacon. Mesenchymal GSCs 3565 were infected using a modified infection protocol [90]. GSCs were dissociated and combined with viral particles at a MOI of 24. Cells were subsequently spun down at 800 g for 1 h at room temperature. Following infection, cells were resuspended and cultured in puromycin (2.1 μg/ml) to select for stably expressing cells. U251 and U343 cells are infected at a MOI of 10. Forty-eight hours following infection, cells were switched to puromycin-containing media (U343: 8 μg/ml; U251 2.1 μg/ml) for 2 weeks.

### GSC culture and analyses of proliferation and differentiation

GSC lines (mesenchymal 83NS, 1123NS, 3565, 3128; proneural 19NS and 84NS) were gifts from Drs. Jeremy Rich, Christopher Hubert, and Ichiro Nakano [22, 91]. GSCs were cultured in serum-free media consisting of Neurobasal-A media supplemented with B-27, Sodium Pyruvate, Glutamax, Pen/Strep, 20 ng/ml EGF (ThermoFisher), and 20 ng/ml hFGF (PeproTech). GSCs were pulsed every 72 h with EGF/FGF. GSCs were disassociated with Accutase (ThermoFisher) at room temperature for 10 min.

GSC lines were dissociated and reversibly transfected at a density of 10^4^ cells/well with siRNA control or siRNA SERBP1 and plated in 96-well plates precoated 3 h prior with Geltrex™ LDEV-Free Reduced Growth Factor Basement Membrane Matrix (19.2–28.8 μg/ml). Growth curves were determined by placing cells in the Incucyte® system (Essen BioSciences, Ann Arbor, MI), an automated and non-invasive method of monitoring live cells in culture. Cells were counted every 4 h, for 7 or more days.

GSCs were differentiated by withdrawing growth factors and culturing GSCs in DMEM/F12 supplemented with 10% FBS and Pen/Strep for 7 days. All experiments were performed with three biological and technical replicates.

### MTS assay

For cell viability assays, 10^4^ cells per well were plated in a 96-well plate and then transfected with siRNA (control or SERBP1). Seventy-two hours after transfection, cell viability was measured using CellTiter-Glo (Promega, Madison, WI) following the manufacturer’s instructions. Absorbance at 490 nm was quantified using the SpectraMax M5 microplate reader (Molecular Devices). All experiments were performed with technical triplicates.

### Cell invasion assay

Invasion assays were performed as described in [92], with a few modifications. A Boyden chamber (CytoSelect 24-well cell invasion assay, Cell Biolabs, San Diego, CA) was used. U251- and U343-transfected cells were harvested using trypsin and counted. Half a million cells were resuspended in serum-free medium. A total of 500 μl of medium containing 10% fetal bovine serum was added to the lower chamber, and 300 μl of resuspended cells was placed in the upper chamber. The assay mixture was incubated at 37 °C and in 5% CO_2_ atmosphere for 18 h. The plate was removed, and the medium inside the insert was aspirated. The chamber was stained with 1% crystal violet. A microscopic image of the insert was taken with a Nikon Eclipse TS100 inverted microscope equipped with a DS-L2 camera control unit (Nikon Instruments, Inc., Melville, NY) at × 20 magnification. Next, crystal violet was dissolved from stained plates using an SDS solution and optical density was measured with a BioTek Synergy HT microplate reader (BioTek, Winooski, VT) at 570 nm. All experiments were performed with technical triplicates.

### Colony formation assay

U251- and U343-transfected cells (siRNA control and SERBP1) were harvested using trypsin and re-plated in 6-well plates (5000 cells/well). Cells were kept in culture for 10–14 days until colonies were clearly visible. Colonies were fixed with 4% paraformaldehyde solution and visualized by staining with 1% crystal violet. Microscopic images were taken with a Nikon Eclipse TS100 inverted microscope equipped with a DS-L2 camera control unit (Nikon Instruments, Melville, NY) at × 20 magnification. Crystal violet was dissolved from stained plates and optical density was measured with a microplate reader at 570 nm. All experiments were performed with technical triplicates.

### Apoptosis assays

Caspase-3/7 activity was measured with the Caspase-Glo 3/7 assay (Promega, Madison, WI), following the manufacturer’s instructions. U251 and U343 cells were reversibly transfected with 25 nM siRNA (control or SERBP1) and seeded in white opaque 96-well plates (10,000 cells/well). Seventy-two hours after transfection, Caspase 3/7 substrate was added to each well and cells were incubated for 1 h. Luminescence was measured with a Molecular Device SpectraMax M5 microplate reader (based on the manufacture’s information). All experiments were performed in triplicate.

### Annexin staining

U251 and U343 cells were reverse transfected (siRNA control and SERBP1) and harvested 72 h later using trypsin. 2.5 × 10^5^ cells were prepared following an Annexin V-FITC kit (catalog #: A13199, V13246 Thermo Fisher). Annexin staining was subsequently measured by flow cytometry. All experiments were performed with biological and technical triplicates.

### Quantification of neurite outgrowth

Neuroblastoma BE-(2)-C cells were obtained from the ATCC and grown in DMEM/F12 (GIBCO) supplemented with 10% fetal bovine serum (GIBCO) and 1% Pen/Strep (GIBCO). BE-(2)-C cells were infected with pUltra-Control or pUltra-SERBP1 virus. Seventy-two hours later, infected cells were plated into 96-well plate at 2000 cells per well and treated with or without 10 μM retinoic acid. Neurite outgrowth was quantified on an IncuCyte ZOOM Imaging System (Essen BioScience) 4 days after platting. For each cell line, a neurite definition was created using the NeuroTrack software module (Essen BioScience). All experiments were performed with biological and technical triplicates.

### Neurosphere formation

U343 pUltra-Control or pUltra-SERBP1 overexpressing cells were trypsinized, washed, and plated at a density of 10 cells/μl in Neurosphere Media consisting of Neurobasal-A supplemented with B-27, N2, Glutamax, Pen/Strep, 50 ng/ml EGF (ThermoFisher), and 50 ng/ml hFGF (PeproTech). Cells were pulsed with growth factors every 72 h. Fourteen days later, neurospheres were quantified and subsequently dissociated with Accutase™, before being replated at the same density (10 cells/μl) to form the next generation of neurospheres. This procedure was repeated for four generations. All experiments were performed with biological and technical triplicates.

### Response to radiation

U343 pUltra-Control or pUltra-SERBP1 overexpressing cells were exposed to various doses (0–10 Gy) of ionizing radiation (IR), using a CP-160 Cabinet X-Radiator (Faxitron X-Ray Corp). Cells were immediately trypsinized and plated into a 24-well plate at a density of 400 cells per well. Two weeks later, cells were fixed with 4% paraformaldehyde for 10 min at room temperature and stained with a 0.5% crystal violet solution in 25% methanol for 20 min. Fixed cells were then washed with H_2_O until excess crystal violet was removed and were dried overnight. Crystal violet was dissolved from colonies using 10% acetic acid for 15 min and optical density was measured with a microplate reader at 590 nm. All experiments were performed in triplicate.

### Response to PRC2 inhibitor

SERBP1 knockdown by siRNA transfection in U251 cells was done as described above. Cells were treated with different concentration of EED226 (MCE MedChemExpress, USA) at the time of siRNA transfection. Cell proliferation was measured using the IncuCyte Live-Cell Analysis System as described above.

### Statistical analysis

Statistical tests were selected based upon data distribution and variance characteristics. Statistical significance was determined using unpaired Student’s *t* test, while Bonferroni correction was used for multiple comparisons. Statistical analyses were carried out in GraphPad Prism 8.3. Significant differences are indicated as follows: ∗*P* ≤ 0.05, ∗∗*P* ≤ 0.01, ∗∗∗*P* ≤ 0.001.

### Intracranial orthotopic xenografts

Stably expressing tetracycline-inducible shSERBP1 3565 GSCs were pre-treated with either vehicle (H_2_O) or doxycycline (1 mg/ml) for 48 h. Upon dissociation, 2.5 × 10^4^ cells in 5 μL of Neurobasal media were implanted orthotopically into the right cerebrum of 6-week-old NCR-SCID mice. Each group contained 10 mice (5 males and 5 females). The following day, water for each group was switched to vehicle (5% sucrose) or doxycycline (5% sucrose + 2 mg/ml doxycycline) (Sigma) and mice were allowed to drink ad libitum. Mice were monitored regularly and euthanized if they displayed neurologic symptoms and/or significant decreases in body weight. Survival differences were assessed with a Kaplan-Meier survival curve and significance with a log-rank test (Mantel–Cox) using Graphpad Prism 8.3. All procedures involving animals in this study were approved by the Institutional Animal Care and Use Committee of UTHSCSA.

### Immunoblotting

Cell pellets were re-suspended and sonicated in Laemmli sample buffer, separated on an 8% or 10% SDS-PAGE gel, and transferred to nitrocellulose membranes. Ten micrograms of protein were loaded into 10% SDS-PAGE gels, followed by transfer to PVDF or nitrocellulose membranes. Membranes were blocked in TBS-T + 5% milk and then probed with the following antibodies: PARP (Cell Signaling Technology), SERBP1 (Bethyl Laboratories), PHGDH (GeneTex), MTHFD2 (GeneTex), PSAT1 (GeneTex), AKT (Cell Signaling Technology), pAKT (Cell Signaling Technology), H3K27me3 (Diagenode), and α-tubulin (Sigma-Aldrich). Horseradish peroxidase (HRP)-conjugated goat anti-rabbit antibody (Santa Cruz Biotechnology) or HRP-conjugated goat anti-mouse antibody (ThermoFisher) was used as a secondary antibody. Bound antibodies were detected using Immobilon Western Chemiluminescent HRP Substrate (Millipore).

### RNA compete assay

#### Protein purification

Full-length SERBP1 coding sequence was cloned into modified pDEST-Magic vector (pTH6838, [23, 93], resulting in an N-terminally-tagged GST-SERBP1 expression construct. This was transformed into *Escherichia coli* C41 cells (Lucigen) and protein expression was induced by adding IPTG (1 mM final) to log phase cell culture and incubating overnight at 16 °C. Cell lysates were prepared by sonication, and then added to GST resin for binding. After washing off non-specific binders, GST-tagged protein was eluted using 250 mM NaCl, 50 mM Tris-HCl (pH 8.8), 30 mM reduced glutathione, 10 mM BME and 20% Glycerol. Protein concentration and purity were estimated by SDS-PAGE and Bradford assay. Details for GST-SERBP1 expression and purification can be found elsewhere [93].

#### The RNA pool generation, RNAcompete pulldown assays, and microarray hybridizations

The RNA pool generation, RNAcompete pulldown assays, and microarray hybridizations were performed as previously described [23, 93, 94]. Briefly, RNAcompete experiments employed defined RNA pools that are generated from 244 K Agilent custom DNA microarrays. Pool design is based on a de Bruijn sequence of order 11 that was subsequently modified to minimize secondary structure in the designed sequences and minimize intramolecular RNA cross-hybridization. After these modifications, not every 11-mer is represented but each 9-mer is represented at least 16 times. To facilitate internal data comparisons, the pool is split computationally into two sets: set A and set B. Each set contains at least 155 copies of all 7-mers except GCTCTTC and CGAGAAG which are removed because they correspond to the SapI/ BspQI restriction site used during DNA template pool generation. A φ2.5 bacteriophage T7 promoter initiating with an AGA or AGG sequence is added at the beginning of each probe sequence in the DNA template pool to enable RNA synthesis. The final RNA pool consists of 241,399 individual sequences up to 41 nucleotides in length (Ray et al. Nature 2013). The microarray design is detailed in [93] and can be ordered from Agilent Technologies using AMADID# 02451. In RNAcompete assays, 20 pmoles of full-length GST-tagged SERBP1 and RNA pool (1.5 nmoles) were incubated in 1 ml of Binding Buffer (20 mM Hepes pH 7.8, 80 mM KCl, 20 mM NaCl, 10% glycerol, 2 mM DTT, 0.1 μg/μL BSA) containing 20 μl glutathione Sepharose 4B (GE Healthcare) beads (pre-washed 3 times in Binding Buffer) for 30 min at 4 °C and subsequently washed four times for 2 min with Binding Buffer at 4 °C. One-sided *Z*-scores were calculated for the motifs as described previously [93].

### Motif analysis RIP-Seq data

To evaluate whether the set of SERBP1 associated transcripts identified by RIP-Seq are enriched for the SERBP1-binding motif in their 3′ UTRs, we performed a permutation analysis (10,000 iterations). In each iteration, we randomly selected a subset of 1293 genes (number of identified SERBP1 targets) from our background set, comprising 16,835 protein-coding genes expressed in at least one sample (count ≥ 2). Next, we calculated the proportion of selected genes presenting the identified motifs in their respective 3′ UTR regions compared to our background set. Finally, we created a histogram to summarize the proportion of gene subsets presenting GC-rich motifs in their 3′ UTR regions.

### Luciferase assay

We used lentiviral vectors from the GOCLONE collection (Sigma-Aldrich) which contain a luciferase reporter and the 3′ UTR of PHGDH, SFN, RASD1, SEMA3B, and GAPDH, to generate stable lines in HeLa cells. 0.8 × 10^4^ cells/well from each line were plated in 96-well plates and incubated for 24 h. Cells were then transfected with a plasmid expressing SBP-SERBP1 or SBP-GST using Lipofectamine 3000 (Thermofisher). Forty-eight hours later, the medium was discarded and cells washed with cold PBS. Dual-luciferase reporter assay (Promega) and GloMAX multidetection system (Promega) were subsequently used according to the manufacturer’s instructions. Each experiment was performed in triplicate.

### SERBP1-RNA interaction

#### Protein purification

The gene encoding the full length human SERBP1 (codon optimized) was cloned into a derivative of pET28 plasmid and the resulting construct was transfected into BL21 (DE3) cells. A single bacterial colony was inoculated into a mini-culture, grown overnight at 37 °C and later transferred to 2 l of terrific broth (TB) media. When the culture reached OD595 = 0.6, IPTG was added. Cells were grown overnight at 18 °C; harvested by centrifugation; resuspended in a buffer containing 25 mM Tris-HCl pH 8.0, 0.5 M NaCl, 5 mM Imidazole, 10% Glycerol, 0.2 mM TCEP, 0.1 mg/ml lysozyme, 2 U/ml DNase I, and a protease inhibitor cocktail (Pierce); and lysed by sonication. The soluble fraction was separated by centrifugation and then passed through a 0.22-μm filter. The SERBP1 protein was purified by successive passage through Ni-affinity and ion-exchange columns using an FPLC system. The His tag was proteolytically removed. Finally, the protein was passed through a size exclusion column in a buffer containing 20 mM Tris-HCl pH 8.0, 0.2 M NaCl, 2.5% glycerol, and 0.1 mM TCEP. The purity of fractions was checked by SDS-PAGE. Fractions with > 95% purity were pooled, concentrated to ~ 5 mg/ml, and used immediately in assays.

#### Fluorescence polarization (FP) assay

5′-Fluoresciene-labeled SERBP1 RNA oligo (5′-GCGCGGG-3′) was synthesized in its 2′-ACE-protected form. The deprotection was done as recommended by the manufacturer (Dharmacon Research). The deprotected RNA was resuspended in a buffer containing 10 mM Tris pH 6.5 and 50 mM NaCl. SERBP1-RNA-binding assay was done in a binding buffer containing 10 mM HEPES pH 7.5, 50 mM KCl. Each reaction sample consisted of 40 μl of 5 nM RNA and increasing concentration of protein from 0 to 1000 nM. Samples were incubated at room temperature before measuring changes in fluorescence polarization (FP) using a Pherastar microplate reader (BMG Labtech). FP values were referenced against a blank buffer to account for background correction. The binding data were fit with one site-specific binding model in GraphPad.

### Metabolite extraction and sample preparation

U251 cells stably expressing tet-inducible shControl or shSERBP1 were plated into 15 cm dishes at a density of 5 × 10^6^ cells. Sixteen hours later, cells were either treated with vehicle (water) or doxycycline (400 ng/ml). Twenty-four hours later, the medium was isolated and frozen in liquid nitrogen. Cells were trypsinized and washed with ice-cold PBS. Cells were centrifuged at 180*g* for 5 min at 4 °C, resuspended, and counted. Cells were counted to ensure that each sample had approximately ~ 18 × 10^6^ cells per replicate. Following an additional wash, cells were suspended in 1 ml of ice-cold PBS and centrifuged. The supernatant was aspirated, and cell pellets were subsequently snap-frozen in liquid nitrogen. The experiment was performed in quadruplicates.

### Chemicals and reagents for metabolic analysis

LC-MS grade methanol, 2-propanol, acetonitrile, ammonium formate, ammonium acetate, chloroform, ACS reagent grade ethanol, butylated hydroxytoluene (BHT), 6 N hydrochloric acid (HCl) solution, and mass spectrometry calibration solutions (Thermo Scientific Pierce LTQ Velos ESI positive and negative ion calibration solutions) were obtained from Fisher Scientific (Pittsburgh, PA, USA). All the aqueous solutions were prepared using ultrapure water (Milli-Q system, Millipore, Billerica, MA).

Intracellular metabolites were extracted by adding 20 μl of BHT solution (5000 μg/ml) and 480 μl of chilled extraction buffer [methanol:water (1:1) with 10 mM ammonium bicarbonate] to frozen cell pellets. The resulting solution was transferred into glass vials containing 500 μl chilled chloroform, vortex for 10 s, and centrifuged at 4750 rpm for 20 min at 4 °C. The supernatant (polar phase) was filtered through a Nanosep 3 K ultra centrifugal device (Pall Co. Port Washington, NY, USA) at 10,000 rpm for 2 h at 4 °C and the resulting filtrate was directly injected for LC-MS analyses. The non-polar phase was evaporated to dryness in a CentriVap refrigerated vacuum concentrator (Labconco, Kansas City, MO, USA) at 4 °C and reconstituted in 100 μl of ethanol:HCl (95:5) for the analysis of redox states of coenzyme Q_10_.

### Metabolomic analysis

Metabolic analyses of polar and non-polar fractions were performed on a Hybrid quadrupole-Orbitrap mass spectrometer (Q Exactive, Thermo Scientific, Waltham, MA, USA) hyphenated with a Thermo Scientific Accela 1250 UHPLC system via electrospray ionization source, simultaneously operating in positive/negative polarity switching ionization mode. The operating MS parameters were the same as described earlier [95]. Two different chromatographic columns were used for comprehensive metabolic analysis: (i) the ZIC-HILIC column [150 × 2.1 mm (3.5 μm, 100 Å)] was used for analyzing amino acids, nucleotides, and redox cofactors; and (ii) the Kinetex C_18_ [150 × 2.1 mm (2.6 μm, 100 Å)] column (Phenomenex, Torrance, CA) was used for analyzing polyamines and redox states of coenzyme Q_10_ (CoQ_10_/CoQ_10_H_2_) as described earlier [96]. To ensure consistency in data acquisition over the entire batch, quality control (QC) samples representing the equivalent concentration of all samples were run every four samples.

All raw MS datasets were processed using Sieve 2.2 (Thermo Fisher Scientific) and features with coefficient of variation (CV) lower than 25% in the QC samples were considered for further analysis. Peaks were scaled according to PQN [97] and features were then mined against the KEGG and the Human Metabolome databases [98]. Putative IDs obtained from the database were confirmed by in house database of accurate masses and retention times generated in our laboratory through the IROA300 4, Mass Spectrometry Metabolite Library of Standards (MSMLS; IROA Technologies, Bolton, MA) using the same experimental conditions as for data acquisition from this dataset. Unsupervised multivariate statistical analysis (principal component analysis, PCA) was carried out in PLS-Toolbox (version 8.7, Eigenvector Research, Manson, WA) in MATLAB (Mathworks, Natick, MA) and performed on all metabolites confirmed by an in-house database. Significant metabolites were selected by the volcano plot based on a 0.4 fold-change threshold. Significant *P* value for visualizing volcano plot data was set to 0.05. Control and SERBP1 knockdown groups were compared using a two-sample *t* test and significance was set at *P* < 0.05.

### Intracellular glutathione measurement

U251 shControl and shSERBP1 cells were seeded into opaque 96-well plates at a density of 5.0 × 10^4^. Sixteen hours later, cells were treated with doxycycline (400 ng/ml) every 24 h. At 24 h and 48 h, intracellular glutathione was measured with the GSH/GSSG-Glo Assay (Promega) with normalization performed to viable cell count.

### Metabolic assays—Seahorse

Mitochondrial respiration was assayed by measuring the oxygen consumption rate (OCR) in control or SERBP1 overexpressing U343 glioblastoma cells using a Seahorse XFe96 Analyzer (Agilent Technologies). Cells were plated at a concentration of 15,000–20,000 cells/well into XF96 Seahorse plates 2 days before the experiment. Media was changed the next day to low serum media (containing 2%FBS) and the experiment was run the next day using XF Base Medium Minimal DMEM (Agilent Technologies) supplemented with 1 mM sodium pyruvate, 5.5 mM glucose, and 2 mM glutamine. Injections were prepared following the manufacturer’s protocol for the Mito Stress Kit (Agilent Technologies). OCR was normalized to confluence using an IncuCyte® ZOOM phase-only processing module (Essen Bioscience).

### Reagents

A complete list of reagents and materials used in this research can be found in Additional file 16.

### Availability of data and materials

The sequencing data for the RNA-Seq and RIP-Seq experiments described in this study are available in the European Nucleotide Archive repository (ENA:PRJEB35774) [99]. All datasets are listed in Additional files 6 and 7.

H3K27me3 ChIP-Seq data of glioblastoma cells were downloaded from the dbGaP repository (study accession: phs001389.v1.p1).

## Supplementary information

**Additional file 1: Supplementary Figures and Legends (Figs. S1-S9).** Contains compiled supplementary figures and legends referenced in the main text.

**Additional file 2: Table S1.** SERBP1 expression in glioma (TCGA) and brain/cortex (GTEx).

**Additional file 3: Table S2.** List of TCGA and CGGA glioma patients.

**Additional file 4: Table S3.** Clinicopathological characteristics of 177 glioma patients from Shanghai Changzheng Hospital, and results of SERBP1 immunostaining.

**Additional file 5: Table S4.** Characteristics of all glioma patients in the cohort from the Shanghai Changzheng Hospital.

**Additional file 6: Table S5.** RIP-seq analysis of 293 T cells transfected with SERBP1-streptag. Sheet 1, summary of results; sheet 2, list of SERBP1 target genes; sheet 3, gene ontology analysis; sheet 4, metabolism terms enriched in the GO analysis; sheet 5, Panther pathways analysis; sheet 6, KEGG pathway analysis.

**Additional file 7: Table S6.** RNA-seq analysis of U251 control vs. SERBP1 knockdown samples. Sheet 1, summary of results; sheet 2, list of genes affected by SERBP1 knockdown; sheet 3, list of ncRNA affected by SERBP1 knockdown; sheet 4, gene ontology analysis of down regulated genes; sheet 5, KEGG pathway analysis of down regulated genes.; sheet 6, gene ontology analysis of up regulated genes; sheet 7, REACTOME pathway analysis of up regulated genes; sheet 8, KEGG pathway analysis of down regulated genes.

**Additional file 8: Table S7.** Results of metabolic analysis U251 control vs. U251 SERBP1 knockdown.

**Additional file 9: Table S8.** Gene expression correlation analysis. Genes showing positive and negative (anti-correlation) with SERBP1 in TCGA GBM samples according to R2. Sheet 1, genes showing positive correlation with SERBP1 in TCGA GBM samples; sheet 2, GO analysis of genes showing positive correlation with SERBP1 in TCGA GBM samples**;** sheet 3, genes showing anti-correlation with SERBP1 in TCGA GBM samples; sheet 4, GO analysis of genes showing anti-correlation with SERBP1 in TCGA GBM samples; sheet 5, comparison between enriched GO terms in genes negatively correlated with SERBP1 vs. genes upregulated upon SERBP1 knockdown.

**Additional file 10: Table S9.** Gene expression correlation analysis. Genes showing positive and negative (anti-correlation) with SERBP1 in brain samples (Kang dataset) according to R2. Sheet 1, genes showing positive correlation with SERBP1 in brain samples; sheet 2, comparison between genes showing positive correlation with SERBP1 in brain and TCGA GBM samples and GO analysis of shared genes; sheet 3, genes negatively correlated with SERBP1 in brain samples; sheet 4, comparison between genes negatively correlated with SERBP1 in brain and TCGA GBM samples and GO analysis of shared genes.

**Additional file 11: Table S10.** Gene set enrichment analysis (GSEA) of genes upregulated upon SERBP1 knockdown. Sheet 1, EZH2 and SUZ12 matches in ChEA 2016 datasets; sheet 2, EZH2 and SUZ12 matches in ENCODE 2015 datasets; sheet 3, genes with SUZ12 binding sites compared to upregulated set in SERBP1 knockdown; sheet 4, all genes with EZH2 binding sites compared to upregulated set in SERBP1 knockdown; sheet 5, H3K27me3 profile in embryonic stem cells; sheet 6, all genes with H3K27me3 sites compared to upregulated set in SERBP1 knockdown sheet 7; overlap all results: EZH2, SUZ12 and H3K27me3.

**Additional file 12: Table S11.** Genes in SERBP1 knockdown upregulated set showing H3K27me3 sites in GBM cells according to [45].

**Additional file 13: Table S12.** Expression analyses of SERBP1 knockdown upregulated set in TCGA GBM vs. LGG and TCGA GBM vs. GTEx brain (cortex) samples.

**Additional file 14: Table S13.** List of primers used for cloning

**Additional file 15: Table S14.** List of primers and probes used in qRT-PCR analyses

**Additional file 16.** Complete list of reagents.

**Additional file 17.** Review history.

## References

[1] Cancer Genome Atlas Research N (2008). Comprehensive genomic characterization defines human glioblastoma genes and core pathways. Nature.

[2] Neelamraju Y, Hashemikhabir S, Janga SC (2015). The human RBPome: from genes and proteins to human disease. J Proteome.

[3] Pereira B, Billaud M, Almeida R (2017). RNA-binding proteins in cancer: old players and new actors. Trends Cancer.

[4] Gerstberger S, Hafner M, Tuschl T (2014). A census of human RNA-binding proteins. Nat Rev Genet.

[5] Brinegar AE, Cooper TA (2016). Roles for RNA-binding proteins in development and disease. Brain Res.

[6] Velasco MX, Kosti A, Penalva LOF, Hernandez G (2019). The diverse roles of RNA-binding proteins in glioma development. Adv Exp Med Biol.

[7] Iwaki S, Yamamura S, Asai M, Sobel BE, Fujii S (1819). Posttranscriptional regulation of expression of plasminogen activator inhibitor type-1 by sphingosine 1-phosphate in HepG2 liver cells. Biochim Biophys Acta.

[8] Mason SD, Joyce JA (2011). Proteolytic networks in cancer. Trends Cell Biol.

[9] Wang T, Xu L, Jia R, Wei J (2017). MiR-218 suppresses the metastasis and EMT of HCC cells via targeting SERBP1. Acta Biochim Biophys Sin Shanghai.

[10] Guo K, Zheng S, Xu Y, Xu A, Chen B, Wen Y (2016). Loss of miR-26a-5p promotes proliferation, migration, and invasion in prostate cancer through negatively regulating SERBP1. Tumour Biol.

[11] Bii VM, Collins CP, Hocum JD, Trobridge GD (2018). Replication-incompetent gammaretroviral and lentiviral vector-based insertional mutagenesis screens identify prostate cancer progression genes. Oncotarget.

[12] Koensgen D, Mustea A, Klaman I, Sun P, Zafrakas M, Lichtenegger W, Denkert C, Dahl E, Sehouli J (2007). Expression analysis and RNA localization of PAI-RBP1 (SERBP1) in epithelial ovarian cancer: association with tumor progression. Gynecol Oncol.

[13] Kunkle BW, Yoo C, Roy D (2013). Reverse engineering of modified genes by Bayesian network analysis defines molecular determinants critical to the development of glioblastoma. PLoS One.

[14] Muto A, Sugihara Y, Shibakawa M, Oshima K, Matsuda T, Nadano D (2018). The mRNA-binding protein Serbp1 as an auxiliary protein associated with mammalian cytoplasmic ribosomes. Cell Biochem Funct.

[15] Ahn JW, Kim S, Na W, Baek SJ, Kim JH, Min K, Yeom J, Kwak H, Jeong S, Lee C (2015). SERBP1 affects homologous recombination-mediated DNA repair by regulation of CtIP translation during S phase. Nucleic Acids Res.

[16] Brown A, Baird MR, Yip MC, Murray J, Shao S. Structures of translationally inactive mammalian ribosomes. Elife. 2018;7. 10.7554/eLife.40486 PMC6226290 30355441

[17] Gracheva E, Dus M, Elgin SC (2009). Drosophila RISC component VIG and its homolog Vig2 impact heterochromatin formation. PLoS One.

[18] Tsui C, Inouye C, Levy M, Lu A, Florens L, Washburn MP, Tjian R (2018). dCas9-targeted locus-specific protein isolation method identifies histone gene regulators. Proc Natl Acad Sci U S A.

[19] Bolger GB (2017). The RNA-binding protein SERBP1 interacts selectively with the signaling protein RACK1. Cell Signal.

[20] Liao L, Park SK, Xu T, Vanderklish P, Yates JR (2008). Quantitative proteomic analysis of primary neurons reveals diverse changes in synaptic protein content in fmr1 knockout mice. Proc Natl Acad Sci U S A.

[21] Consortium GT (2013). The genotype-tissue expression (GTEx) project. Nat Genet.

[22] Hubert CG, Rivera M, Spangler LC, Wu Q, Mack SC, Prager BC, Couce M, McLendon RE, Sloan AE, Rich JN (2016). A three-dimensional organoid culture system derived from human glioblastomas recapitulates the hypoxic gradients and cancer stem cell heterogeneity of tumors found in vivo. Cancer Res.

[23] Ray D, Ha KCH, Nie K, Zheng H, Hughes TR, Morris QD (2017). RNAcompete methodology and application to determine sequence preferences of unconventional RNA-binding proteins. Methods.

[24] Mi H, Dong Q, Muruganujan A, Gaudet P, Lewis S, Thomas PD (2010). PANTHER version 7: improved phylogenetic trees, orthologs and collaboration with the Gene Ontology Consortium. Nucleic Acids Res.

[25] Supek F, Bosnjak M, Skunca N, Smuc T (2011). REVIGO summarizes and visualizes long lists of gene ontology terms. PLoS One.

[26] Szklarczyk D, Gable AL, Lyon D, Junge A, Wyder S, Huerta-Cepas J, Simonovic M, Doncheva NT, Morris JH, Bork P (2019). STRING v11: protein-protein association networks with increased coverage, supporting functional discovery in genome-wide experimental datasets. Nucleic Acids Res.

[27] Newman AC, Maddocks ODK (2017). One-carbon metabolism in cancer. Br J Cancer.

[28] Martinez-Outschoorn UE, Peiris-Pages M, Pestell RG, Sotgia F, Lisanti MP (2017). Cancer metabolism: a therapeutic perspective. Nat Rev Clin Oncol.

[29] Pavlova NN, Thompson CB (2016). The emerging hallmarks of cancer metabolism. Cell Metab.

[30] Locasale JW, Grassian AR, Melman T, Lyssiotis CA, Mattaini KR, Bass AJ, Heffron G, Metallo CM, Muranen T, Sharfi H (2011). Phosphoglycerate dehydrogenase diverts glycolytic flux and contributes to oncogenesis. Nat Genet.

[31] Liu J, Guo S, Li Q, Yang L, Xia Z, Zhang L, Huang Z, Zhang N (2013). Phosphoglycerate dehydrogenase induces glioma cells proliferation and invasion by stabilizing forkhead box M1. J Neuro-Oncol.

[32] Tibbetts AS, Appling DR (2010). Compartmentalization of mammalian folate-mediated one-carbon metabolism. Annu Rev Nutr.

[33] Nilsson R, Jain M, Madhusudhan N, Sheppard NG, Strittmatter L, Kampf C, Huang J, Asplund A, Mootha VK (2014). Metabolic enzyme expression highlights a key role for MTHFD2 and the mitochondrial folate pathway in cancer. Nat Commun.

[34] Dai J, Wei R, Zhang P, Kong B (2019). Overexpression of microRNA-195-5p reduces cisplatin resistance and angiogenesis in ovarian cancer by inhibiting the PSAT1-dependent GSK3beta/beta-catenin signaling pathway. J Transl Med.

[35] Liu B, Jia Y, Cao Y, Wu S, Jiang H, Sun X, Ma J, Yin X, Mao A, Shang M (2016). Overexpression of phosphoserine aminotransferase 1 (PSAT1) predicts poor prognosis and associates with tumor progression in human esophageal squamous cell carcinoma. Cell Physiol Biochem.

[36] Vie N, Copois V, Bascoul-Mollevi C, Denis V, Bec N, Robert B, Fraslon C, Conseiller E, Molina F, Larroque C (2008). Overexpression of phosphoserine aminotransferase PSAT1 stimulates cell growth and increases chemoresistance of colon cancer cells. Mol Cancer.

[37] Yang Y, Wu J, Cai J, He Z, Yuan J, Zhu X, Li Y, Li M, Guan H (2015). PSAT1 regulates cyclin D1 degradation and sustains proliferation of non-small cell lung cancer cells. Int J Cancer.

[38] Locasale JW (2013). Serine, glycine and one-carbon units: cancer metabolism in full circle. Nat Rev Cancer.

[39] Ye C, Tu BP (2018). Sink into the epigenome: histones as repositories that influence cellular metabolism. Trends Endocrinol Metab.

[40] Cavuoto P, Fenech MF (2012). A review of methionine dependency and the role of methionine restriction in cancer growth control and life-span extension. Cancer Treat Rev.

[41] Palanichamy K, Thirumoorthy K, Kanji S, Gordon N, Singh R, Jacob JR, Sebastian N, Litzenberg KT, Patel D, Bassett E (2016). Methionine and kynurenine activate oncogenic kinases in glioblastoma, and methionine deprivation compromises proliferation. Clin Cancer Res.

[42] Casero RA, Murray Stewart T, Pegg AE (2018). Polyamine metabolism and cancer: treatments, challenges and opportunities. Nat Rev Cancer.

[43] Guaras A, Perales-Clemente E, Calvo E, Acin-Perez R, Loureiro-Lopez M, Pujol C, Martinez-Carrascoso I, Nunez E, Garcia-Marques F, Rodriguez-Hernandez MA (2016). The CoQH2/CoQ ratio serves as a sensor of respiratory chain efficiency. Cell Rep.

[44] Wang Y, Hekimi S (2016). Understanding ubiquinone. Trends Cell Biol.

[45] Hall AW, Battenhouse AM, Shivram H, Morris AR, Cowperthwaite MC, Shpak M, Iyer VR (2018). Bivalent chromatin domains in glioblastoma reveal a subtype-specific signature of glioma stem cells. Cancer Res.

[46] Santos MC, Tegge AN, Correa BR, Mahesula S, Kohnke LQ, Qiao M, Ferreira MA, Kokovay E, Penalva LO (2016). miR-124, -128, and -137 orchestrate neural differentiation by acting on overlapping gene sets containing a highly connected transcription factor network. Stem Cells.

[47] Spangle JM, Dreijerink KM, Groner AC, Cheng H, Ohlson CE, Reyes J, Lin CY, Bradner J, Zhao JJ, Roberts TM, Brown M (2016). PI3K/AKT signaling regulates H3K4 methylation in breast cancer. Cell Rep.

[48] Najafi M, Mortezaee K, Majidpoor J (2019). Cancer stem cell (CSC) resistance drivers. Life Sci.

[49] Hanahan D, Weinberg RA (2011). Hallmarks of cancer: the next generation. Cell.

[50] Strickland M, Stoll EA (2017). Metabolic reprogramming in glioma. Front Cell Dev Biol.

[51] Maddocks OD, Labuschagne CF, Adams PD, Vousden KH (2016). Serine metabolism supports the methionine cycle and DNA/RNA methylation through de novo ATP synthesis in cancer cells. Mol Cell.

[52] Xiao L, Yang X, Loh YP (2019). Neurotrophic, gene regulation, and cognitive functions of carboxypeptidase E-neurotrophic factor-alpha1 and its variants. Front Neurosci.

[53] Horing E, Harter PN, Seznec J, Schittenhelm J, Buhring HJ, Bhattacharyya S, von Hattingen E, Zachskorn C, Mittelbronn M, Naumann U (2012). The “go or grow” potential of gliomas is linked to the neuropeptide processing enzyme carboxypeptidase E and mediated by metabolic stress. Acta Neuropathol.

[54] Armento A, Ilina EI, Kaoma T, Muller A, Vallar L, Niclou SP, Kruger MA, Mittelbronn M, Naumann U (2017). Carboxypeptidase E transmits its anti-migratory function in glioma cells via transcriptional regulation of cell architecture and motility regulating factors. Int J Oncol.

[55] Ilina EI, Armento A, Sanchez LG, Reichlmeir M, Braun Y, Penski C, Capper D, Sahm F, Jennewein L, Harter PN (2017). Effects of soluble CPE on glioma cell migration are associated with mTOR activation and enhanced glucose flux. Oncotarget.

[56] Sun X, Zhang H, Luo L, Zhong K, Ma Y, Fan L, Fu D, Wan L (2016). Comparative proteomic profiling identifies potential prognostic factors for human clear cell renal cell carcinoma. Oncol Rep.

[57] Tuo L, Xiang J, Pan X, Hu J, Tang H, Liang L, Xia J, Hu Y, Zhang W, Huang A (2019). PCK1 negatively regulates cell cycle progression and hepatoma cell proliferation via the AMPK/p27(Kip1) axis. J Exp Clin Cancer Res.

[58] Liu MX, Jin L, Sun SJ, Liu P, Feng X, Cheng ZL, Liu WR, Guan KL, Shi YH, Yuan HX, Xiong Y (2018). Metabolic reprogramming by PCK1 promotes TCA cataplerosis, oxidative stress and apoptosis in liver cancer cells and suppresses hepatocellular carcinoma. Oncogene.

[59] Tang Y, Zhang Y, Wang C, Sun Z, Li L, Cheng S, Zhou W (2018). Overexpression of PCK1 gene antagonizes hepatocellular carcinoma through the activation of gluconeogenesis and suppression of glycolysis pathways. Cell Physiol Biochem.

[60] Ho PC, Bihuniak JD, Macintyre AN, Staron M, Liu X, Amezquita R, Tsui YC, Cui G, Micevic G, Perales JC (2015). Phosphoenolpyruvate is a metabolic checkpoint of anti-tumor T cell responses. Cell.

[61] Xiong Z, Xiao W, Bao L, Xiong W, Xiao H, Qu Y, Yuan C, Ruan H, Cao Q, Wang K (2019). Tumor cell “slimming” regulates tumor progression through PLCL1/UCP1-mediated lipid browning. Adv Sci (Weinh).

[62] Parkhitko AA, Jouandin P, Mohr SE, Perrimon N (2019). Methionine metabolism and methyltransferases in the regulation of aging and lifespan extension across species. Aging Cell.

[63] Romani M, Pistillo MP, Banelli B (2018). Epigenetic targeting of glioblastoma. Front Oncol.

[64] Danishuddin, Subbarao N, Faheem M, Khan SN: Polycomb repressive complex 2 inhibitors: emerging epigenetic modulators. Drug Discov Today 2019, 24:179–188. 10.1016/j.drudis.2018.07.002 30031878

[65] O'Donnell WT, Warren ST (2002). A decade of molecular studies of fragile X syndrome. Annu Rev Neurosci.

[66] Long K, Moss L, Laursen L, Boulter L, Ffrench-Constant C (2016). Integrin signalling regulates the expansion of neuroepithelial progenitors and neurogenesis via Wnt7a and Decorin. Nat Commun.

[67] Jarvinen TA, Prince S (2015). Decorin: a growth factor antagonist for tumor growth inhibition. Biomed Res Int.

[68] Ma HI, Hueng DY, Shui HA, Han JM, Wang CH, Lai YH, Cheng SY, Xiao X, Chen MT, Yang YP. Intratumoral decorin gene delivery by AAV vector inhibits brain glioblastomas and prolongs survival of animals by inducing cell differentiation. Int J Mol Sci. 2014;15:4393–414. 10.3390/ijms15034393 PMC3975403 24625664

[69] Kim S, Burette A, Chung HS, Kwon SK, Woo J, Lee HW, Kim K, Kim H, Weinberg RJ, Kim E (2006). NGL family PSD-95-interacting adhesion molecules regulate excitatory synapse formation. Nat Neurosci.

[70] Dias CM, Punetha J, Zheng C, Mazaheri N, Rad A, Efthymiou S, Petersen A, Dehghani M, Pehlivan D, Partlow JN (2019). Homozygous missense variants in NTNG2, encoding a presynaptic netrin-G2 adhesion protein, lead to a distinct neurodevelopmental disorder. Am J Hum Genet.

[71] Heimer G, van Woerden GM, Barel O, Marek-Yagel D, Kol N, Munting JB, Borghei M, Atawneh OM, Nissenkorn A, Rechavi G, et al. Netrin-G2 dysfunction causes a Rett-like phenotype with areflexia. Hum Mutat. 2020;41(2):476–86. 10.1002/humu.23945. 10.1002/humu.23945 31692205

[72] Abu-Libdeh B, Ashhab M, Shahrour M, Daana M, Dudin A, Elpeleg O, Edvardson S, Harel T (2019). Homozygous frameshift variant in NTNG2, encoding a synaptic cell adhesion molecule, in individuals with developmental delay, hypotonia, and autistic features. Neurogenetics.

[73] Lin CL, Kong Q, Cuny GD, Glicksman MA (2012). Glutamate transporter EAAT2: a new target for the treatment of neurodegenerative diseases. Future Med Chem.

[74] Rothstein JD, Dykes-Hoberg M, Pardo CA, Bristol LA, Jin L, Kuncl RW, Kanai Y, Hediger MA, Wang Y, Schielke JP, Welty DF (1996). Knockout of glutamate transporters reveals a major role for astroglial transport in excitotoxicity and clearance of glutamate. Neuron.

[75] Wang J, Hjelmeland AB, Nabors LB, King PH (2019). Anti-cancer effects of the HuR inhibitor, MS-444, in malignant glioma cells. Cancer Biol Ther.

[76] Yi C, Li G, Ivanov DN, Wang Z, Velasco MX, Hernandez G, Kaundal S, Villarreal J, Gupta YK, Qiao M (2018). Luteolin inhibits Musashi1 binding to RNA and disrupts cancer phenotypes in glioblastoma cells. RNA Biol.

[77] Li H, Handsaker B, Wysoker A, Fennell T, Ruan J, Homer N, Marth G, Abecasis G, Durbin R (2009). Genome project data processing S: the sequence alignment/map format and SAMtools. Bioinformatics.

[78] Bray NL, Pimentel H, Melsted P, Pachter L (2016). Near-optimal probabilistic RNA-seq quantification. Nat Biotechnol.

[79] Soneson C, Love MI, Robinson MD (2015). Differential analyses for RNA-seq: transcript-level estimates improve gene-level inferences. F1000Res.

[80] Tang Z, Li C, Kang B, Gao G, Li C, Zhang Z (2017). GEPIA: a web server for cancer and normal gene expression profiling and interactive analyses. Nucleic Acids Res.

[81] Frankish A, Diekhans M, Ferreira AM, Johnson R, Jungreis I, Loveland J, Mudge JM, Sisu C, Wright J, Armstrong J (2019). GENCODE reference annotation for the human and mouse genomes. Nucleic Acids Res.

[82] Love MI, Huber W, Anders S (2014). Moderated estimation of fold change and dispersion for RNA-seq data with DESeq2. Genome Biol.

[83] Penalva LO, Keene JD: Biotinylated tags for recovery and characterization of ribonucleoprotein complexes. Biotechniques 2004, 37:604, 606, 608–610. 10.2144/04374ST05 15517973

[84] Jupe S, Fabregat A, Hermjakob H: Expression data analysis with Reactome. Curr Protoc Bioinformatics 2015, 49:8 20 21–28 20 29. 10.1002/0471250953.bi0820s49 PMC4407007 25754994

[85] Kuleshov MV, Jones MR, Rouillard AD, Fernandez NF, Duan Q, Wang Z, Koplev S, Jenkins SL, Jagodnik KM, Lachmann A (2016). Enrichr: a comprehensive gene set enrichment analysis web server 2016 update. Nucleic Acids Res.

[86] Consortium F, The RP, Clst, Forrest AR, Kawaji H, Rehli M, Baillie JK, de Hoon MJ, Haberle V, Lassmann T, et al: A promoter-level mammalian expression atlas. Nature 2014, 507:462–470. 10.1038/nature13182 PMC4529748 24670764

[87] Quinlan AR, Hall IM (2010). BEDTools: a flexible suite of utilities for comparing genomic features. Bioinformatics.

[88] Bowman RL, Wang Q, Carro A, Verhaak RG, Squatrito M (2017). GlioVis data portal for visualization and analysis of brain tumor expression datasets. Neuro-Oncology.

[89] Tiscornia G, Singer O, Verma IM (2006). Production and purification of lentiviral vectors. Nat Protoc.

[90] Berggren WT, Lutz M, Modesto V: General Spinfection Protocol. In StemBook. Cambridge: Harvard Stem Cell Institute; 2008. 23658993

[91] Mao P, Joshi K, Li J, Kim SH, Li P, Santana-Santos L, Luthra S, Chandran UR, Benos PV, Smith L (2013). Mesenchymal glioma stem cells are maintained by activated glycolytic metabolism involving aldehyde dehydrogenase 1A3. Proc Natl Acad Sci U S A.

[92] Uren PJ, Vo DT, de Araujo PR, Potschke R, Burns SC, Bahrami-Samani E, Qiao M, de Sousa AR, Nakaya HI, Correa BR (2015). RNA-binding protein Musashi1 is a central regulator of adhesion pathways in glioblastoma. Mol Cell Biol.

[93] Ray D, Kazan H, Cook KB, Weirauch MT, Najafabadi HS, Li X, Gueroussov S, Albu M, Zheng H, Yang A (2013). A compendium of RNA-binding motifs for decoding gene regulation. Nature.

[94] Ray D, Kazan H, Chan ET, Pena Castillo L, Chaudhry S, Talukder S, Blencowe BJ, Morris Q, Hughes TR (2009). Rapid and systematic analysis of the RNA recognition specificities of RNA-binding proteins. Nat Biotechnol.

[95] Lu X, Solmonson A, Lodi A, Nowinski SM, Sentandreu E, Riley CL, Mills EM, Tiziani S (2017). The early metabolomic response of adipose tissue during acute cold exposure in mice. Sci Rep.

[96] Pandey R, Riley CL, Mills EM, Tiziani S (2018). Highly sensitive and selective determination of redox states of coenzymes Q9 and Q10 in mice tissues: application of orbitrap mass spectrometry. Anal Chim Acta.

[97] Dieterle F, Ross A, Schlotterbeck G, Senn H (2006). Probabilistic quotient normalization as robust method to account for dilution of complex biological mixtures. Application in 1H NMR metabonomics. Anal Chem.

[98] Wishart DS, Tzur D, Knox C, Eisner R, Guo AC, Young N, Cheng D, Jewell K, Arndt D, Sawhney S (2007). HMDB: the human Metabolome database. Nucleic Acids Res.

[99] Kosti A, de Araujo PR, Li WQ, Guardia G, Chiou J, Yi C, Ray D, Meliso F, Li YM, Delambre T, Qiao M, Burns SS, Lorbeer FK, Georgi F, Flosbach M, Klinnert S, Jennseit A, Lei X, Sandoval CR, Ha K, Zheng H, Pandey R, Gruslova A, Gupta Y, Brenner A, Kokovay E, Hughes TR, Morris Q, Galante P, Tiziani S, Penalva LOF. The RNA binding protein SERBP1 functions as a novel oncogenic factor in glioblastoma by bridging cancer metabolism and epigenetic regulation. RNA sequencing of U251 SERBP1 knockdown cells and RIP sequencing for the identification of SERBP1 targets in 293T cells. European Nucleotide Archive: PRJEB35774. http://www.ebi.ac.uk/ena/browser/view/PRJEB35774 (2020).

[100] Louis DN, Perry A, Reifenberger G, von Deimling A, Figarella-Branger D, Cavenee WK, Ohgaki H, Wiestler OD, Kleihues P, Ellison DW. The 2016 World Health Organization classification of tumors of the central nervous system: a summary. Acta Neuropathol. 2016;(131):803–20. 10.1007/s00401-016-1545-1 27157931

