# Supplementary material for: The RNA-binding protein SERBP1 functions as a novel oncogenic factor in glioblastoma by bridging cancer metabolism and epigenetic regulation
Source: Genome Biol. 2020 Aug 6;21:195. doi: 10.1186/s13059-020-02115-y (PMC7412812; doi:10.1186/s13059-020-02115-y)
Supplement: Supplementary file 1 — Additional file 1: Supplementary Figures and Legends (Figs. S1-S9). Contains compiled supplementary figures and legends referenced in the main text. [file 13059_2020_2115_MOESM1_ESM.pdf]

The RNA binding protein SERBP1 functions as a novel oncogenic factor in glioblastoma by bridging cancer metabolism and epigenetic regulation

Additional File 1

Supplementary Figures

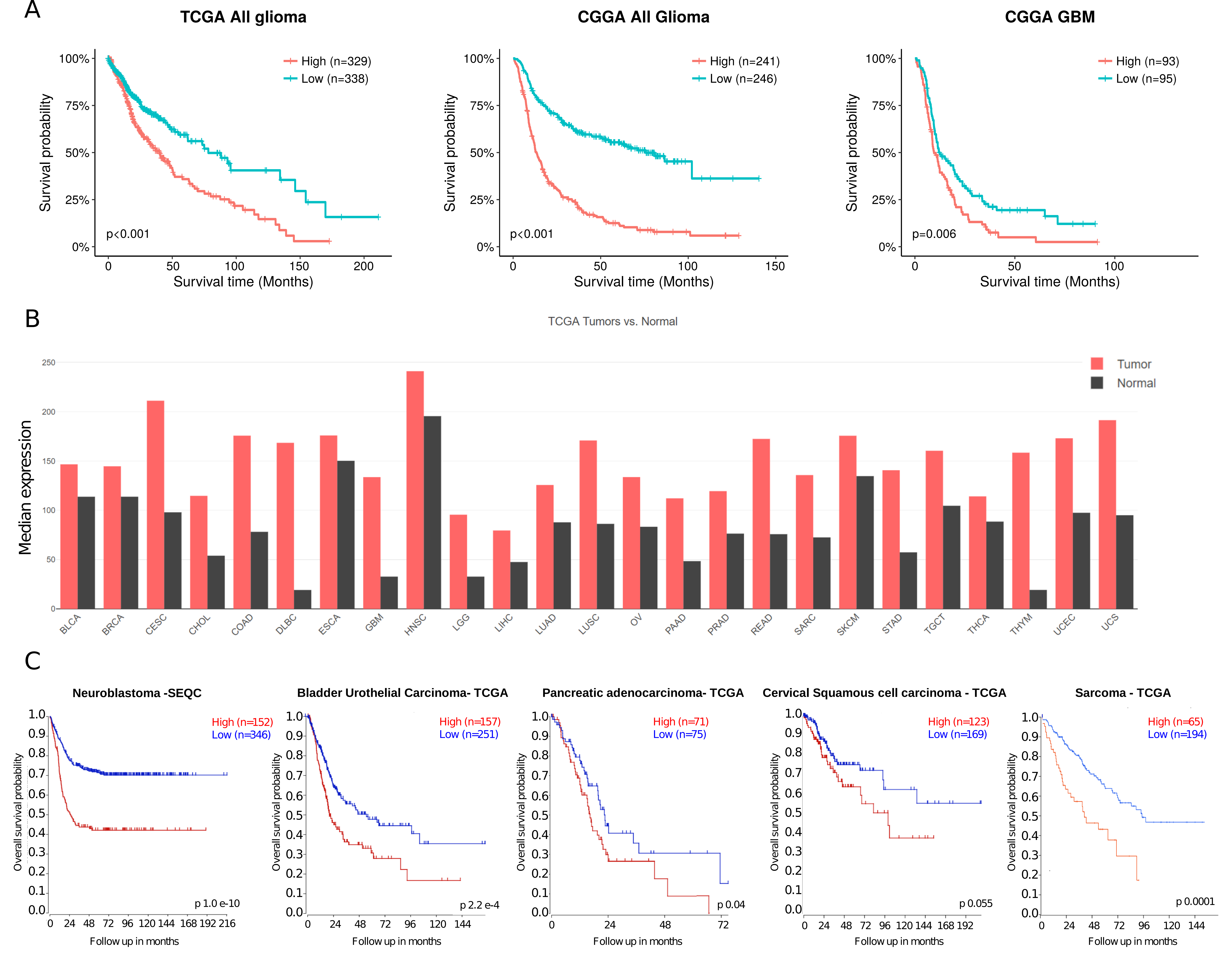
A
TCGA All glioma
High (n=329)
Low (n=338)
Survival probability
p<0.001
Survival time (Months)
CGGA All Glioma
High (n=241)
Low (n=246)
Survival probability
p<0.001
Survival time (Months)
CGGA GBM
High (n=93)
Low (n=95)
Survival probability
p=0.006
Survival time (Months)
B
TCGA Tumors vs. Normal
Tumor
Normal
Median expression
BLCA
BRCA
CESC
CHOL
COAD
DLBC
ESCA
GBM
HNSC
LGG
LIHC
LUAD
LUSC
OV
PAAD
PRAD
READ
SARC
SKCM
STAD
TGCT
THCA
THYM
UCEC
UCS
C
Neuroblastoma -SEQC
High (n=152)
Low (n=346)
Overall survival probability
p 1.0 e-10
Follow up in months
Bladder Urothelial Carcinoma- TCGA
High (n=157)
Low (n=251)
Overall survival probability
p 2.2 e-4
Follow up in months
Pancreatic adenocarcinoma- TCGA
High (n=71)
Low (n=75)
Overall survival probability
p 0.04
Follow up in months
Cervical Squamous cell carcinoma - TCGA
High (n=123)
Low (n=169)
Overall survival probability
p 0.055
Follow up in months
Sarcoma - TCGA
High (n=65)
Low (n=194)
Overall survival probability
p 0.0001
Follow up in months

Fig. S1. SERBP1 expression in normal vs. normal tissue and impact on survival. A) Kaplan–Meier curves show the survival of 667 glioma patients in the TCGA cohort, 487 glioma patients in the CGGA cohort and 188 glioblastoma patients in the CGGA cohort displaying low and high SERBP1 levels. **B)** SERBP1 expression in tumor (TCGA) vs. normal tissue (GTEx). Data was generated using Gepia [24]. ACC-Adrenocortical carcinoma, BLCA-Bladder Urothelial Carcinoma, BRCA-Breast invasive carcinoma, CESC-Cervical squamous cell carcinoma and endocervical adenocarcinoma, CHOL-Cholangio carcinoma, COAD-Colon adenocarcinoma, DLBC-Lymphoid Neoplasm Diffuse Large B-cell Lymphoma, ESCA-Esophageal carcinoma, GBM-GBM multiforme, HNSC-Head and Neck squamous cell carcinoma, LGG-Lower Grade Glioma, LIHC-Liver hepatocellular carcinoma, LUAD-Lung adenocarcinoma, LUSC-Lung squamous cell carcinoma, OV-Ovarian serous cystadenocarcinoma, PAAD-Pancreatic adenocarcinoma, PRAD-Prostate adenocarcinoma, READ-Rectum adenocarcinoma, SARC-Sarcoma, SKCM-Skin Cutaneous Melanoma, STAD-Stomach adenocarcinoma, TGCT-Testicular Germ Cell Tumors, THCA-Thyroid carcinoma, THYM-Thymoma, UCEC-Uterine Corpus Endometrial Carcinoma, UCS-Uterine Carcinosarcoma. **C)** Examples of tumor types in which high expression levels of SERBP1 are associated with a poorer prognosis. Datasets were obtained from R2 Genomics Analysis and Visualization Platform. Kaplan–Meier curves indicate the survival of patients displaying low and high SERBP1 levels; default parameters were used, Kaplan Scan (KaplanScan) established optimum survival cut-off based on statistical testing.

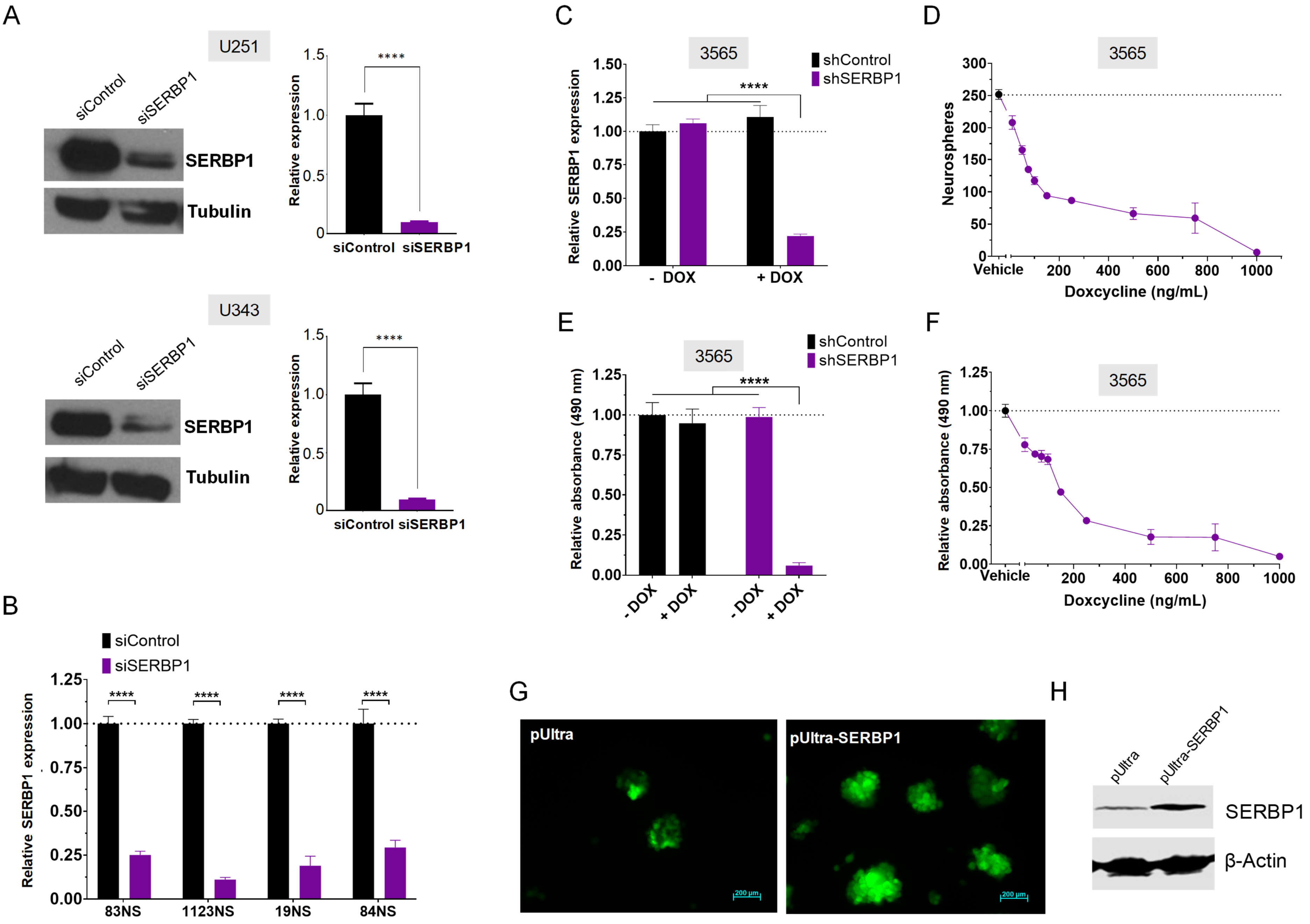
A
U251
siControl
siSERBP1
SERBP1
Tubulin
Relative expression
siControl
siSERBP1
U343
B
siControl
siSERBP1
Relative SERBP1 expression
83NS
1123NS
19NS
84NS
C
3565
shControl
shSERBP1
Relative SERBP1 expression
- DOX
+ DOX
D
Neurospheres
Vehicle
Doxycline (ng/mL)
E
Relative absorbance (490 nm)
F
G
pUltra
pUltra-SERBP1
H
β-Actin

Fig. S2. SERBP1 expression in knockdown and over expressing cell lines used in the study. **A)** Western shows SERBP1 knockdown levels 72 hours post siRNA transfection in U251 and U343 cells. **B)** SERBP1 knockdown levels 72 hours post siRNA transfection in different GSC lines was measured by qRT-PCR. **C)** 3565 shControl and shSERBP1 GSC lines were exposed to Doxycycline to induce shRNA expression and SERBP1 knockdown levels were evaluated 24 hours later by qRT-PCR. **D)** Reduction in 3565 shSERBP1 neurosphere formation was proportional to the amount of Doxycycline used to trigger SERBP1 knockdown. **E)** 3565 shControl and shSERBP1 GSC lines were exposed to Doxycycline to induce shRNA expression; impact of SERBP1 reduced expression on cell viability was measured by MTS. **F)** Reduction in 3565 shSERBP1 viability was proportional to the amount of Doxycycline used to trigger SERBP1 knockdown. **G)** Aspect of control and SERBP1 Overexpression (OE) U343 cells grown as neurospheres. **H)** Western showing levels of SERBP1 expression in control (pUltra) and SERBP1 OE lines.

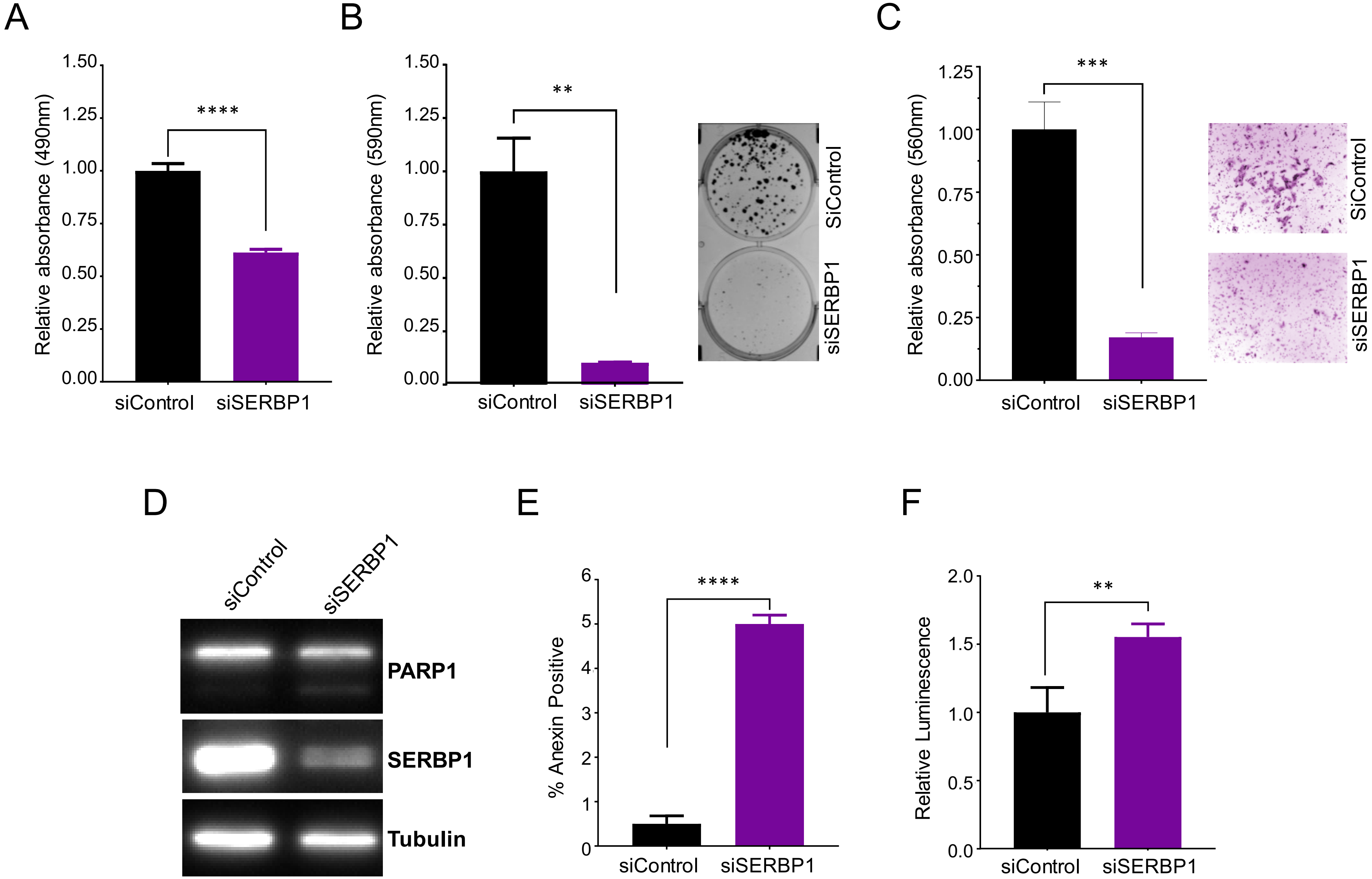
A
B
C
D
E
F
Relative absorbance (490nm)
1.50
1.25
1.00
0.75
0.50
0.25
0.00
****
siControl
siSERBP1
Relative absorbance (590nm)
**
SiControl
siSERBP1
Relative absorbance (560nm)
***
siControl
siSERBP1
PARP1
SERBP1
Tubulin
% Anexin Positive
6
5
4
3
2
1
0
****
Relative Luminescence
2.0
1.5
1.0
0.5
0.0
**

Fig. S3. SERBP1 affects cancer related phenotypes. A) Knockdown of SERBP1 in U343 cells decreased viability (MTS assay). **B)** SERBP1 KD in U343 cells diminished clonogenic potential, as measured by colony formation assay. **C)** The Boyden chamber assay was used to evaluate SERBP1 impact on invasion; values of crystal violet absorbance showed that SERBP1 KD decreased invasion potential. **D-E)** SERBP1 silencing increased apoptosis as indicated by PARP1 cleavage (D) and Annexin staining (E). Data were analyzed with Students' t-test and presented as mean $\pm$ standard deviation. Bonferroni-correction was used for multiple-comparisons. * = $p \leq 0.05$; ** = $p \leq 0.01$; *** = $p \leq 0.001$; **** $p \leq 0.0001$.

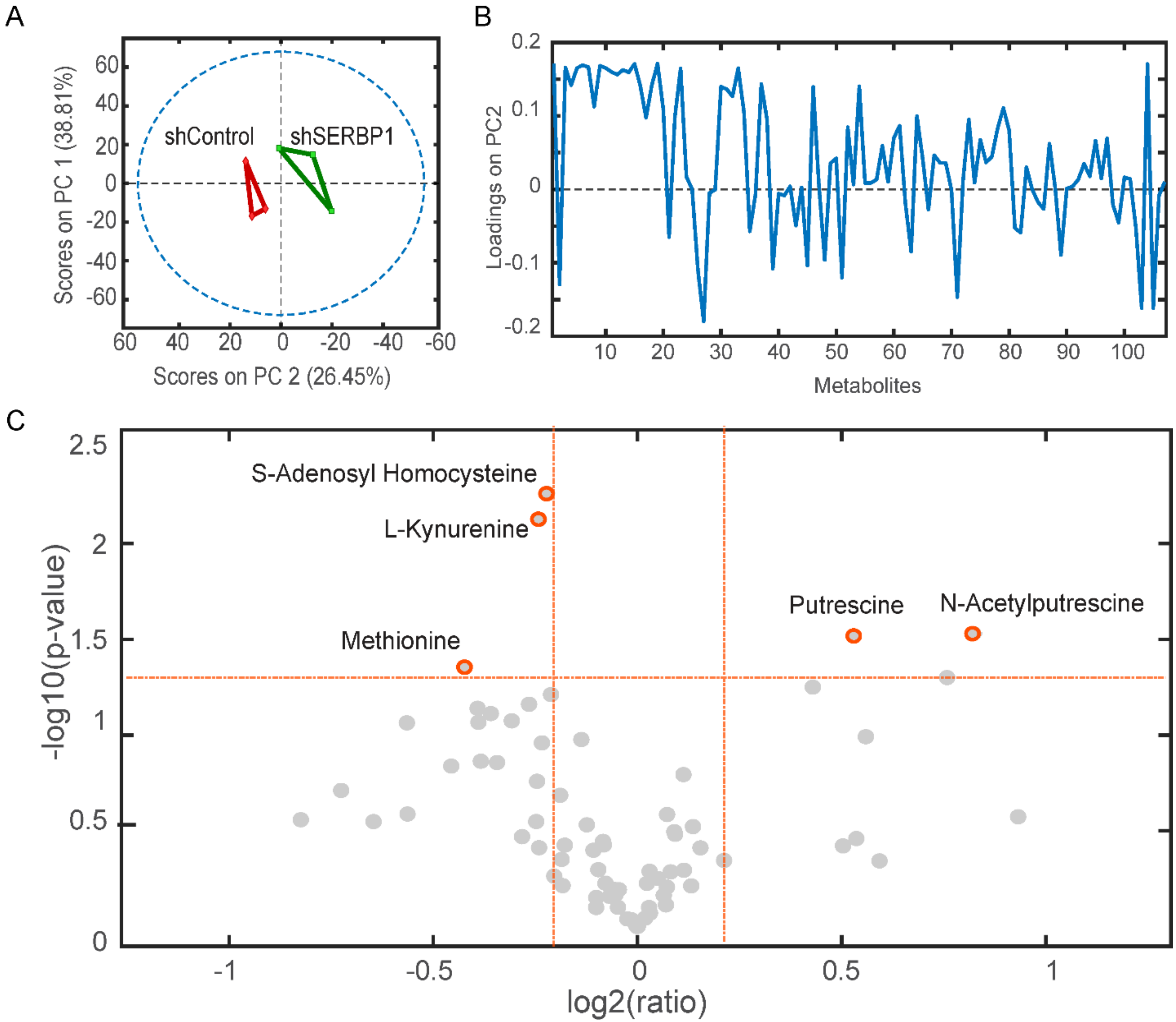
A
B
C
shControl
shSERBP1
Scores on PC 1 (38.81%)
Scores on PC 2 (26.45%)
Loadings on PC2
Metabolites
S-Adenosyl Homocysteine
L-Kynurenine
Methionine
Putrescine
N-Acetylputrescine
-log10(p-value)
log2(ratio)

Fig. S4. Metabolomic analysis of the impact of SERBP1 knockdown. A) Principal Component Analysis (PCA) score plot performed on validated metabolites assigned using in-house small molecule compounds library, shows a distinct separation when comparing control to silenced SERBP1 groups. **B)** The loadings plot for principle component 2 shows the contribution of several metabolites to the variation between groups. **C)** The volcano plot highlights statistically significant metabolites which are shown in metabolomics data (Fig. 5). Statistical significance was set to p= 0.05, fold change=0.4.

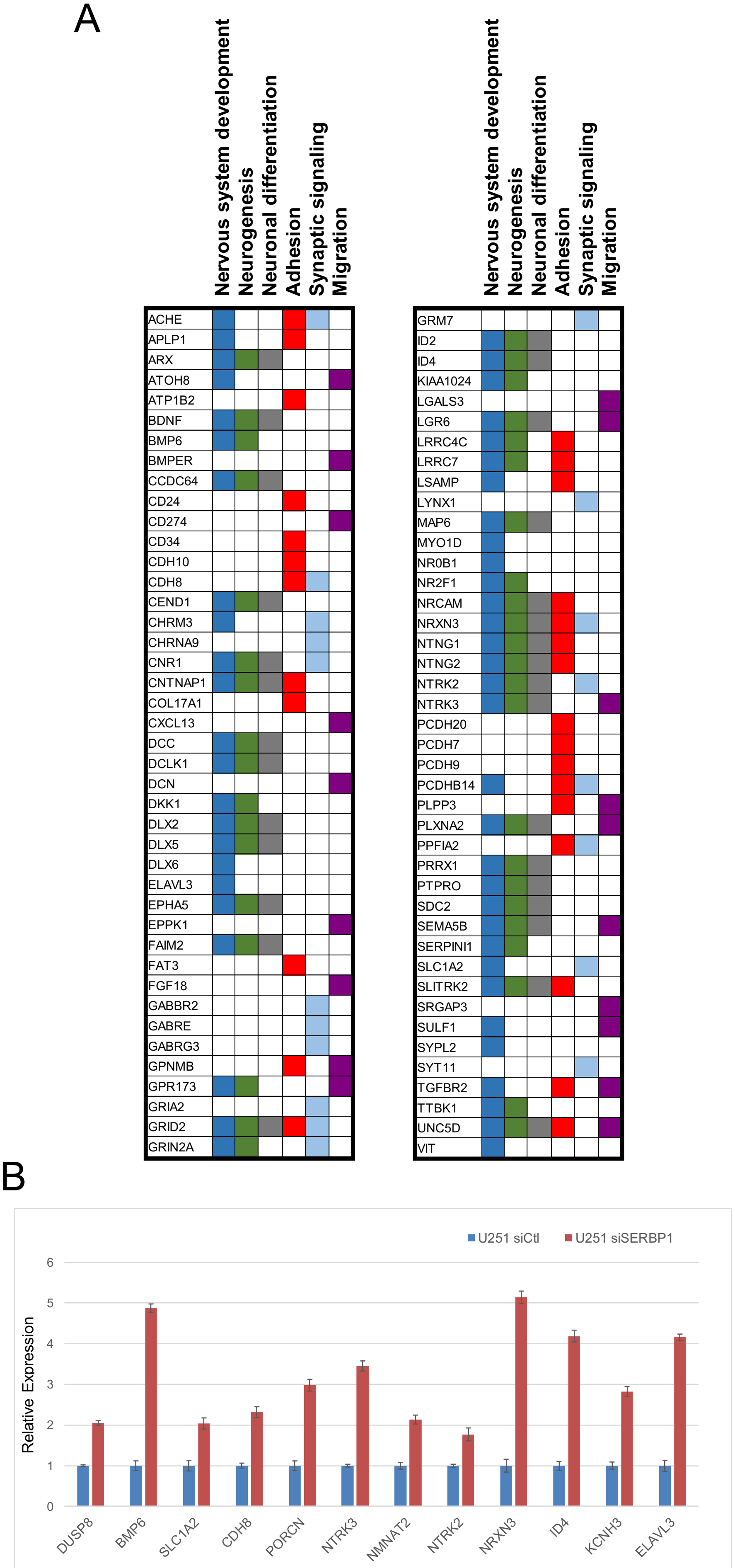
A
Nervous system development
Neurogenesis
Neuronal differentiation
Adhesion
Synaptic signaling
Migration
ACHE
APLP1
ARX
ATOH8
ATP1B2
BDNF
BMP6
BMPER
CCDC64
CD24
CD274
CD34
CDH10
CDH8
CEND1
CHRM3
CHRNA9
CNR1
CNTNAP1
COL17A1
CXCL13
DCC
DCLK1
DCN
DKK1
DLX2
DLX5
DLX6
ELAVL3
EPHA5
EPPK1
FAIM2
FAT3
FGF18
GABBR2
GABRE
GABRG3
GPNMB
GPR173
GRIA2
GRID2
GRIN2A
GRM7
ID2
ID4
KIAA1024
LGALS3
LGR6
LRRC4C
LRRC7
LSAMP
LYNX1
MAP6
MYO1D
NR0B1
NR2F1
NRCAM
NRXN3
NTNG1
NTNG2
NTRK2
NTRK3
PCDH20
PCDH7
PCDH9
PCDHB14
PLPP3
PLXNA2
PPFIA2
PRRX1
PTPRO
SDC2
SEMA5B
SERPINI1
SLC1A2
SLITRK2
SRGAP3
SULF1
SYPL2
SYT11
TGFBR2
TTBK1
UNC5D
VIT
B
U251 siCtl
U251 siSERBP1
Relative Expression
0
1
2
3
4
5
6
DUSP8
BMP6
SLC1A2
CDH8
PORCN
NTRK3
NMNAT2
NTRK2
NRXN3
ID4
KCNH3
ELAVL3

Fig. S5. Upregulated genes in SERBP1 knockdown cells. A) Main biological functions associated with genes upregulated in SERBP1 knockdown U251 cells. **B)** Validation of expression variation induced by SERBP1 knockdown by qRT-PCR.

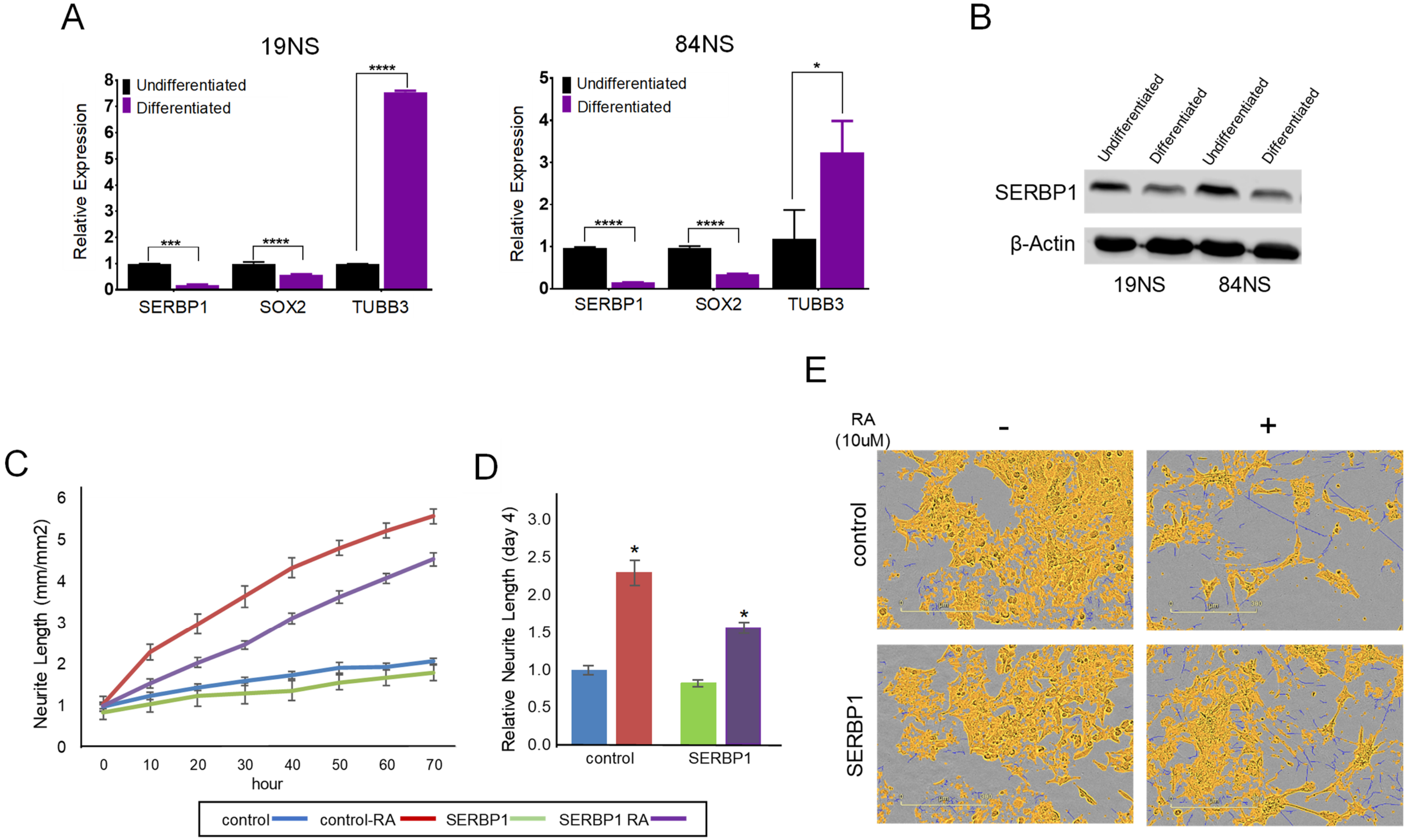
A
19NS
Undifferentiated
Differentiated
Relative Expression
SERBP1
SOX2
TUBB3
***
****
****
84NS
*
B
Undifferentiated
Differentiated
Undifferentiated
Differentiated
SERBP1
β-Actin
19NS
84NS
C
Neurite Length (mm/mm2)
hour
control
control-RA
SERBP1
SERBP1 RA
D
Relative Neurite Length (day 4)
control
SERBP1
E
RA
(10uM)
-
+
control
SERBP1

Fig. S6. SERBP1 promotes "stemness". A-B) GSC lines were transferred to differentiation media and SERBP1 expression levels in undifferentiated and differentiated cells were measured by (A) qRT-PCR and (B) Western blots. Sox2 and Tubb3 were used as markers of stem and differentiated cells, respectively. **C-E)** BE(2)C neuroblastoma cells were infected with control or SERBP1 expression lentivirus or control and treated with or without 10 μM retinoic acid. IncuCyte was used to measure neurite outgrowth as a marker of neuronal differentiation. (C) Length of neurites over time. (D) Untreated and retinoic acid-treated cells at 70 hours post-treatment showing effects of SERBP1 on neuronal differentiation. (E) Aspects of control and SERBP1 OE cells at 0 and 70 hour post-treatment. Neurites are highlighted in blue.

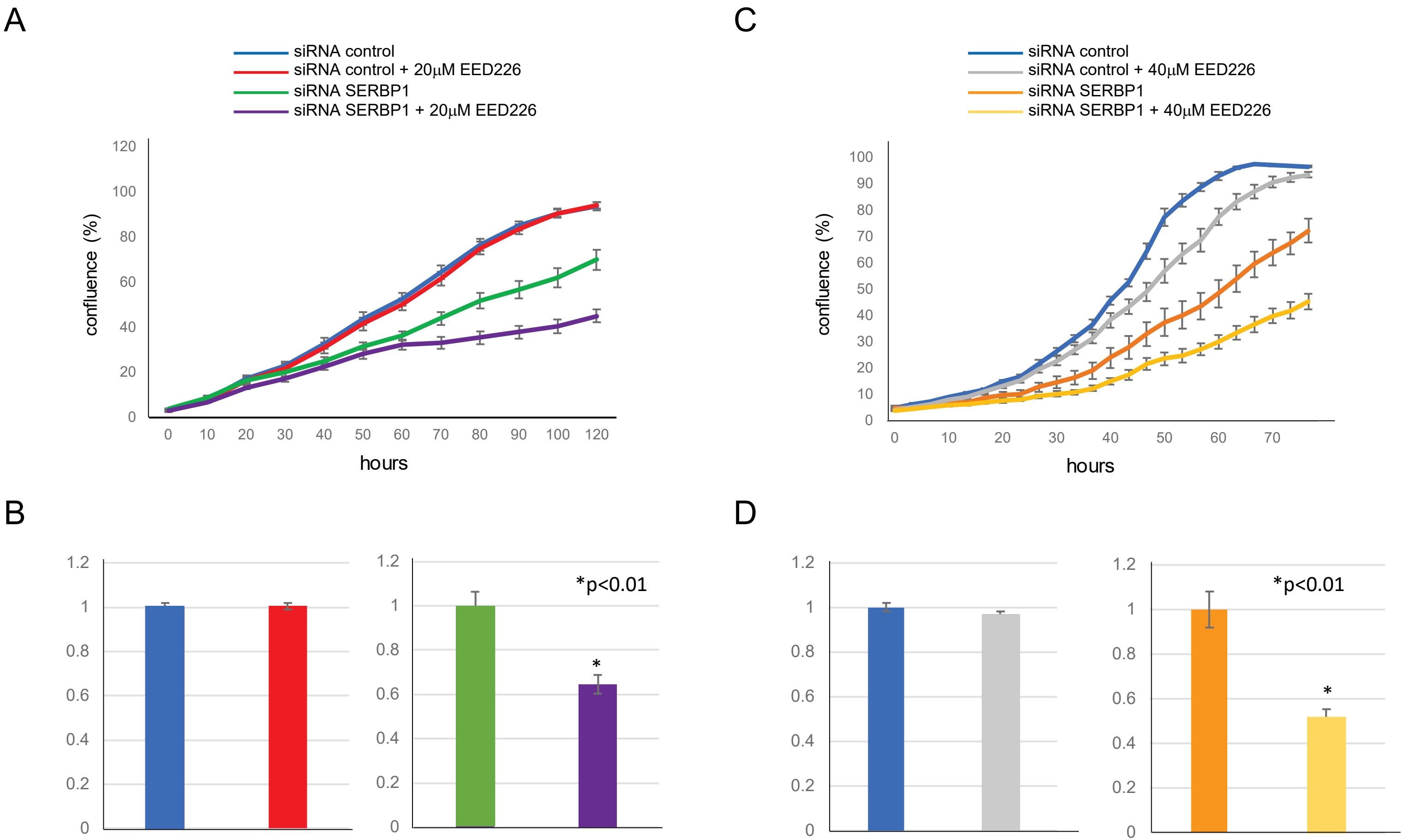
A
siRNA control
siRNA control + 20μM EED226
siRNA SERBP1
siRNA SERBP1 + 20μM EED226
confluence (%)
hours
0
10
20
30
40
50
60
70
80
90
100
120
B
*p<0.01
*
C
siRNA control
siRNA control + 40μM EED226
siRNA SERBP1
siRNA SERBP1 + 40μM EED226
confluence (%)
hours
D
*p<0.01
*

Fig. S7. SERBP1 knockdown increases sensitivity to PRC2 inhibitor, EED226. U251 cells were transfected with control or SERBP1 siRNA and treated with PRC2 inhibitor EED226 to determine if a reduction in SERBP1 expression increases sensitivity to the drug. **A)** Proliferation curve obtained with Incucyte; cells were treated with 20 µM EED226. **B)** Graph displays difference in proliferation between control and treated cells at a single time point (120 h). **C)** Proliferation curve obtained with Incucyte, cells were treated with 40 µM EED226. **D)** Differences in proliferation between control and treated cells at 70 hours.

A

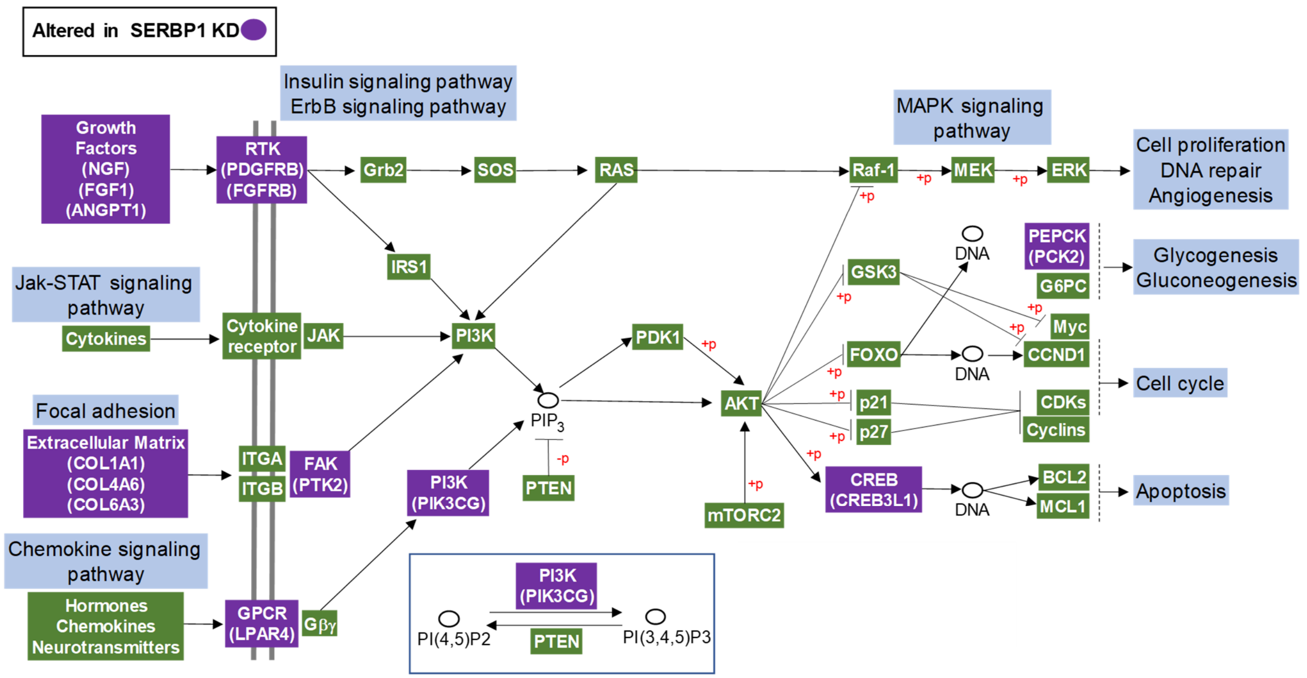

B

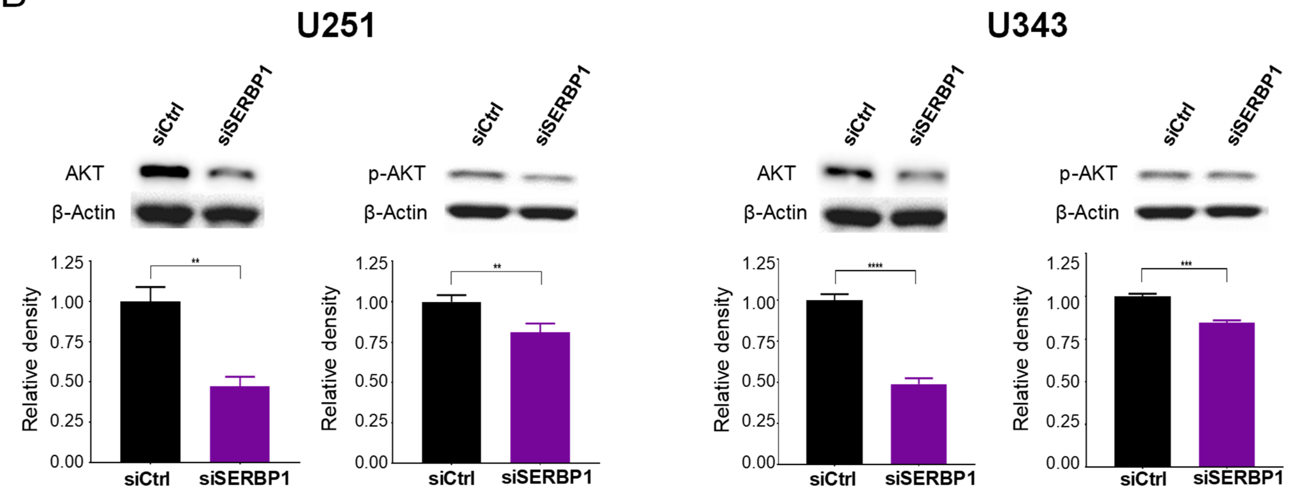

C

		SERBP1 staining intensity scores in glioma patients				
		-	+	++	+++	
		N=38	N=32	N=66	N=41	Total
AKT1 staining intensity scores in glioma patients	-	24	15	15	4	58
	+	8	11	9	4	32
	++	6	5	28	7	46
	+++	0	1	14	26	41

P<0.001

Fig. S8. SERBP1's impact on the AKT pathway. A) KEGG AKT pathway; genes showing expression changes after SERBP1 knockdown in U251 cells are shown in purple. **B)** Western analysis showing that SERBP1 knockdown affected AKT and p-AKT levels in U251 and U343 cells. **C)** Association of AKT1 staining intensity scores levels with SERBP1 staining intensity scores using pairwise combinations in samples from all patients with GBM in the Shanghai Changzheng Hospital cohort (n=177).

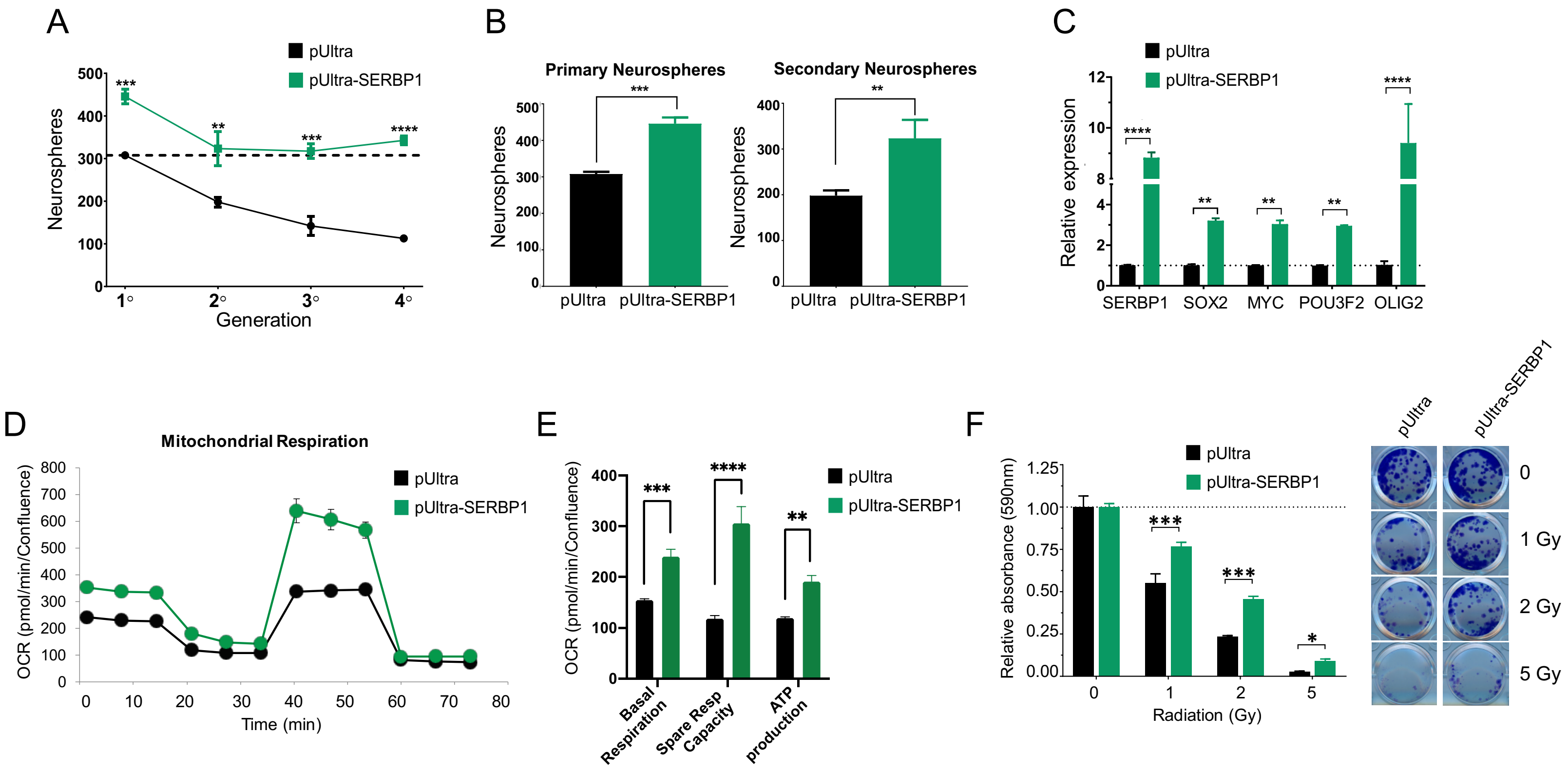
A
pUltra
pUltra-SERBP1
Neurospheres
Generation
1° 2° 3° 4°
B
Primary Neurospheres
Secondary Neurospheres
Neurospheres
pUltra pUltra-SERBP1
C
Relative expression
SERBP1 SOX2 MYC POU3F2 OLIG2
D
Mitochondrial Respiration
OCR (pmol/min/Confluence)
Time (min)
E
Basal Respiration
Spare Resp Capacity
ATP production
F
Relative absorbance (590nm)
Radiation (Gy)
0
1 Gy
2 Gy
5 Gy

Fig. S9. SERBP1 over expression promotes “glioma stem cell’ phenotypes. A) U343 control (pUltra) and U343 SERBP1 OE lines were grown in stem cell media. The graph shows the number of neurospheres in passages 1-4. **B)** Graphs show the number of primary and secondary neurospheres of control and U343 SERBP1 OE lines. **C)** Expression of stem cell markers in neurospheres of control and SERBP1 OE lines. **D)** Graphs show mitochondria respiration in control and U343 SERBP1 OE cells measured over time with a Seahorse XFe96. **E)** Graphs show basal respiration, and ATP production, spare respiration capacity in control and U343 SERBP1 OE cells measured with a Seahorse XFe96. **F)** U343 control (pUltra) and U343 SERBP1 OE lines were exposed to increased amount of radiation and the impact on cell proliferation was measured via colony formation assay. Left: relative absorbance (590nm) as measurement of colony quantification. Right: aspect of plates. Data were analyzed with Student’s t-test and presented as mean $\pm$ standard deviation. Bonferroni-correction was used for multiple-comparisons. * = $p \leq 0.05$; ** = $p \leq 0.01$; *** = $p \leq 0.001$; **** $p \leq 0.0001$.
