# Supplementary material for: The RNA-binding protein SERBP1 functions as a novel oncogenic factor in glioblastoma by bridging cancer metabolism and epigenetic regulation
Source: Genome Biol. 2020 Aug 6;21:195. doi: 10.1186/s13059-020-02115-y (PMC7412812; doi:10.1186/s13059-020-02115-y)
Supplement: Supplementary file 2 — Additional file 2: Table S1. SERBP1 expression in glioma (TCGA) and brain/cortex (GTEx). [file 13059_2020_2115_MOESM2_ESM.pdf]

	samples	median	mad	mean	sd
Cortex	464 (41.13%)	22.14	6.49	23.10	7.67
Grade II	248 (21.99%)	82.22	29.03	89.16	33.30
Grade III	262 (23.23%)	104.90	43.97	109.38	45.79
Grade IV	154 (13.65%)	126.82	44.24	123.87	44.46
TOTAL	1128	62.59	58.73	71.42	52.56
