# Supplementary material for: The RNA-binding protein SERBP1 functions as a novel oncogenic factor in glioblastoma by bridging cancer metabolism and epigenetic regulation
Source: Genome Biol. 2020 Aug 6;21:195. doi: 10.1186/s13059-020-02115-y (PMC7412812; doi:10.1186/s13059-020-02115-y)
Supplement: Supplementary file 4 — Additional file 4: Table S3. Clinicopathological characteristics of 177 glioma patients from Shanghai Changzheng Hospital, and results of SERBP1 immunostaining. [file 13059_2020_2115_MOESM4_ESM.docx]

| **Variables** | **N(%)** |
| --- | --- |
| **Gender Male** | 101(57.1) |
| **Age (years) ≥55** | 109(61.6) |
|  |  |
| **Tumor site** |  |
| **Temporal lobe** | 28 (15.8) |
| **Parietal lobe** | 76 (42.9) |
| **Frontal lobe** | 49 (27.7) |
| **Occipital lobe** | 24 (13.6) |
| **Tumor size** |  |
| **>4 cm** | 101(57.1) |
| **Extent of resection** |  |
| **Total** | 154(87.0) |
| **Subtotal** | 23 (13.0) |
| **Radiotherapy (GBM patients) Yes** | 54 (45.8) |
| **Chemotherapy (GBM patients) Yes** | 83 (70.3) |
|  |  |
| **WHO grade^*^** |  |
| **I** | 14 (7.9) |
| **II** | 27 (15.3) |
| **III** | 18 (10.2) |
| **IV** | 118(66.7) |

^*^Tumors were graded according to the 2016 World Health Organization (WHO) criteria

| **Staining intensity scores of SERBP1** | | | | | | |
| --- | --- | --- | --- | --- | --- | --- |
|  | -  (n=38) | +  (n=32) | ++  (n=66) | +++  (n=41) | Total | *P-value* |
| **WHO grade**  **I**  **II**  **III**  **IV** |  |  |  |  |  | <0.001 |
|  | 10  12 | 3  4 | 1  8 | 0  3 | 14  27 |  |
|  | 3 | 4 | 8 | 3 | 18 |  |
|  | 13 | 21 | 49 | 35 | 118 |  |

|  | **Low SERBP1**  **expression** | **High SERBP1**  **expression** | Total | *P-value* |
| --- | --- | --- | --- | --- |
| **WHO grade**  **I**  **II**  **III**  **IV** |  |  |  | <0.001 |
|  | 13 | 1 | 14 |  |
|  | 16 | 11 | 27 |  |
|  | 7  34 | 11  84 | 18  118 |  |

|  | **Low SERBP1 expression** | **High SERBP1**  **expression** | Total | *P-value* |
| --- | --- | --- | --- | --- |
| **Grade**  **Low**  **High** |  |  |  | 0.002 |
|  | 29 | 12 | 41 |  |
|  | 41 | 95 | 136 |  |
