# Supplementary material for: The RNA-binding protein SERBP1 functions as a novel oncogenic factor in glioblastoma by bridging cancer metabolism and epigenetic regulation
Source: Genome Biol. 2020 Aug 6;21:195. doi: 10.1186/s13059-020-02115-y (PMC7412812; doi:10.1186/s13059-020-02115-y)
Supplement: Supplementary file 17 — Additional file 17. Review history. [file 13059_2020_2115_MOESM17_ESM.docx]

Review History

**First round of review**

**Reviewer 1**

**Are you able to assess all statistics in the manuscript, including the appropriateness of statistical tests used?**

There are no statistics in the manuscript.

**Comments to author:**

In this manuscript, Kosti et al reported that RNA binding protein SERBP1 is a novel regulator of GBM, which controls cancer metabolism and indirectly modulates epigenetic regulation. First of all, the point that SERBP1 contributes to tumor development has been reported in many cancer types like: ovarian cancer (DOI:10.1016/j.ygyno.2007.06.023); prostate cancer(DOI:10.1007/s13277-016-5158-z); (doi: 10.1093/abbs/gmx017.); Hepatocellular carcinoma (doi: 10.1093/abbs/gmx017.). Thus it is easy to reason that it may also function in GBM, which greatly jeopardize the novelty of the finding. Moreover, there are a lot of flaws in study design.

1.     In Figure 2, only one set of siRNA was used to silence the expression of SERBP1. More than two sets of siRNAs should be used.
2.     In each survival figure(for instance Figure 1D-H, Figure 2I), please show the number of cases in each group in the graph.
3.     In vivo experiment, "Cells were treated with doxycycline prior to implantation and mice were allowed to drink water containing doxycycline ad libitum. " . Technically, cells were weakened before implantation. Doxycycline will be very hard to get through the blood brain barrier, which makes it almost impossible to be utilized in clinical treatment in the treatment of GBM.
4.     In Figure 2, to prove that SERBP1 affects cancer-related phenotypes and tumor growth, more in vivo experiments are needed.
5.     Overall, the link between SERBP1 and cancer metabolism as well as that between SERBP1 and epigenetic control are superficially touched upon for each part without in-depth mechanistic exploration.
6.     In figure 5, the point that SERBP1 impacts on one-carbon and methyl cycles and potential downstream effects failed to show its relation with GBM development. Perhaps it is a universal mechanism for almost any types of cancer cells.
7.     No supplementary Figure 5 and supplementary Figure 6 were found.
8.     While this study initially analyzed the tissue-specific expression of SERBP1, it lacks a genome-wide screening or identification process of this protein, which somewhat makes this study fall a little bit out of Genome Biology publication scope.

In general, I would suggest the manuscript to go to a more specialized journal.

**Reviewer 2**

**Are you able to assess all statistics in the manuscript, including the appropriateness of statistical tests used?**

No, I do not feel adequately qualified to assess the statistics.

**Comments to author:**

The authors have identified an RNA binding protein SERBP1 as a novel regulator of glioblastoma (GBM) proliferation and metabolism. Using extensive bioinformatic, genomic and metabolomic analyses, the authors show that SERBP1 regulates one carbon metabolism and methionine levels. SERBP1 downregulation restricts methionine availability and H3K27me3 methylation, thereby altering epigenetic silencing and adversely impacting GBM growth. This is an impressive, well-crafted and novel manuscript that merits publication in Genome Biology. I have a few minor suggestions that need to be addressed as detailed below:

1)     Did the authors perform an unbiased principal component analysis of their metabolomic data? Clearly there is a lot of metabolic data in their supplementary material and I feel the quality of the manuscript will be improved if an unbiased analysis is done.
2)     Did the authors examine any patient-derived GBM models? The U251 and U343 lines have been in culture for a long time and are poor models of the in vivo disease.
3)      Did the authors measure glutathione levels by any complementary methods other than their mass spectrometry analysis? They show that cysteine levels are significantly reduced in shSERBP1 cells, and claim that "since cysteine is a precursor of glutathione (GSH), a redox regulating metabolite, its depletion might affect the redox balance in SERPB1 knockdown cells (Fig. 5B).". This is an important point- however I don't see an alteration in glutathione levels in Supplementary table 7.
4)     Can the authors explain why there is no difference in cystathionine levels following  SERBP1 silencing? Since homocysteine levels are altered, I would have expected to see a difference in cystathionine levels.
5)     The logic behind investigating the PI3K/Akt pathway in SERBP1 knockdown cells is not clear. There are other pathways that control the GBM epigenome. Can the authors please clarify?
6)     The authors state that "SERBP1 OE cells also had increased mitochondrial respiration and ATP production compared to controls (Additional File 1: Fig. S8D-E). But I didn't see a clear difference in ATP levels following SERBP1 knockdown (Supp table 7). Can the authors explain the disconnect?

**Authors Response**

**Point-by-point responses to the reviewers’ comments:**

Reviewer #1
1. In this manuscript, Kosti et al reported that RNA binding protein SERBP1 is a novel regulator of GBM, which controls cancer metabolism and indirectly modulates epigenetic regulation. First of all, the point that SERBP1 contributes to tumor development has been reported in many cancer types like: ovarian cancer (DOI:10.1016/j.ygyno.2007.06.023); prostate cancer(DOI:10.1007/s13277-016-5158-z); (doi: 10.1093/abbs/gmx017.); Hepatocellular carcinoma (doi: 10.1093/abbs/gmx017.). Thus it is easy to reason that it may also function in GBM, which greatly jeopardize the novelty of the finding.
*The reviewer describes SERBP1 participation in other tumors almost suggesting we ignored this information. In fact, we listed all these articles in the introduction. Moreover, we have a figure showing SERBP1 expression in numerous tumor types and survival curves in other tumors. The fact that SERBP1 was previously implicated in other tumor types does not take away the novelty of our manuscript. None of these articles describes SERBP1 targets and mechanism of action in tumorigenesis. Our article is novel since we described for the first time an RBP functioning as a critical regulator of metabolism and SERBP1 impact on epigenetic regulation. In reality, the fact that SERBP1 plays a role in other tumor types just makes our findings more relevant.*

2.In Figure 2, only one set of siRNA was used to silence the expression of SERBP1. More than two sets of siRNAs should be used.
*This statement is incorrect. We used a smart-pool (4 different siRNA at equimolar ratios), which reduces off-target effects. Additionally, we used a tet-inducible shRNA with a different sequence to conduct other assays and it produced the same phenotype as the smart-pool.*

3.In each survival figure(for instance Figure 1D-H, Figure 2I), please show the number of cases in each group in the graph.
*Detailed patient information is provided in supplementary files and we mentioned the number of cases in the figure legend.*

4.In vivo experiment, "Cells were treated with doxycycline prior to implantation and mice were allowed to drink water containing doxycycline ad libitum. " . Technically, cells were weakened before implantation. Doxycycline will be very hard to get through the blood brain barrier, which makes it almost impossible to be utilized in clinical treatment in the treatment of GBM.
*The statement is incorrect and the comment on the use of Doxycycline to treat GBM patients suggests that the reviewer did not understand the design of the experiment.
We opted to use a tet-inducible system to knockdown SERBP1 in the xenograft experiment since SERBP1 KO or stable KD lines are not viable. Doxycycline and tet-inducible systems are often used in neuroscience studies [1, 2], and glioblastoma mouse models [3, 4]. Additionally, the blood brain barrier is leaky and compromised in glioblastoma resulting in better drug penetration [5].
A study examining the CNS penetration of doxycycline in mice with intact blood brain barriers found that daily 100 mg/kg doses resulted in brain doxycycline concentrations ranging from 857ng/mL to 1396 ng/mL [6] , which would be more than enough to inhibit GSC growth (Figure S2).
In the xenograft study our mice drank approximately ~3 ml per day, which at 2mg/ml of doxycycline would result in 6 mg per day. Based on our average mouse weight (~26g), our mice were receiving approximately 230 mg/kg, which would mean that doxycycline levels in the brain are more than enough to activate the shRNA against SERBP1, which is supported by the survival study results. The initial Dox pulse performed before implantation serve to activate the shRNA expression and it is a common practice in this type of experiments.
We have never suggested the potential use of Doxycycline or the system used to knockdown SERBP1 to treat glioblastoma patients. Reviewer 1 comment is puzzling. In fact, we discuss that SERBP1 targeting requires the identification of a small molecule inhibitor.

1. Ramirez S, Liu X, Lin PA, Suh J, Pignatelli M, Redondo RL, Ryan TJ, Tonegawa S: Creating a false memory in the hippocampus. Science 2013, 341:387-391.
2. Tayler KK, Tanaka KZ, Reijmers LG, Wiltgen BJ: Reactivation of neural ensembles during the retrieval of recent and remote memory. Curr Biol 2013, 23:99-106.
3. Braun CJ, Stanciu M, Boutz PL, Patterson JC, Calligaris D, Higuchi F, Neupane R, Fenoglio S, Cahill DP, Wakimoto H, et al: Coordinated Splicing of Regulatory Detained Introns within Oncogenic Transcripts Creates an Exploitable Vulnerability in Malignant Glioma. Cancer Cell 2017, 32:411-426 e411.
4. Park JW, Wollmann G, Urbiola C, Fogli B, Florio T, Geley S, Klimaschewski L: Sprouty2 enhances the tumorigenic potential of glioblastoma cells. Neuro Oncol 2018, 20:1044-1054.
5. Chacko AM, Li C, Pryma DA, Brem S, Coukos G, Muzykantov V: Targeted delivery of antibody-based therapeutic and imaging agents to CNS tumors: crossing the blood-brain barrier divide. Expert Opin Drug Deliv 2013, 10:907-926.
6. Lucchetti J, Fracasso C, Balducci C, Passoni A, Forloni G, Salmona M, Gobbi M: Plasma and Brain Concentrations of Doxycycline after Single and Repeated Doses in Wild-Type and APP23 Mice. J Pharmacol Exp Ther 2019, 368:32-40.*

5. In Figure 2, to prove that SERBP1 affects cancer-related phenotypes and tumor growth, more in vivo experiments are needed.
*Once again, the reviewer did not provide any rationale and did not elaborate on why more in vivo experiments are needed and what type of in vivo experiments are missing. We provide plenty of data in Figures 1, 2, S2, S3, S5 and S8 that supports SERBP1 impact on cancer-related phenotypes. The* *experiments show that 1) SERBP1 high expression is prevalent in GBM and associated with poor prognosis and response to therapy, 2) SERBP1 knockdown in two GBM cell lines impacted survival, colony formation, apoptosis and invasion, 3) SERBP1 knockdown in five GSC lines showed that SERBP1 affects their proliferation and survival, 4) SERBP1 knockdown also affected differentiation of GSCs and neuroblastoma cells and expression of stem cell markers, 5) SERBP1 increased expression promoted stemness and boosted radio-resistance, 6) SERBP1 knockdown in xenograft model increased survival and affected tumor growth.*

6.Overall, the link between SERBP1 and cancer metabolism as well as that between SERBP1 and epigenetic control are superficially touched upon for each part without in-depth mechanistic exploration.
*As before, either the reviewer overlooked experiments or was not capable of following our experimental strategy. Our model is supported by an extensive amount of data. Recapitulating, we showed that SERBP1 knockdown affects the expression of genes implicated in One-carbon and Methyl cycles by RNA-seq. These changes were corroborated by qRT-PCR, western and immuno-staining. Metabolomic analysis corroborated the impact of SERBP1 on One-carbon and Methyl cycles. In particular, we observed a decrease in methionine production. We investigate then, if this effect could compromise histone methylation. We showed by Western that SERBP1 knockdown decreases H3K27me3 levels. In agreement, we established that SERBP1 knockdown increases sensitivity to a PRC2 inhibitor. Finally, we indicated that genes upregulated in SERBP1 knockdown cells, which are strongly associated with neuronal differentiation and function, tend to be methylated (H3K23me3 sites) in GBM cells.*

7.In figure 5, the point that SERBP1 impacts on one-carbon and methyl cycles and potential downstream effects failed to show its relation with GBM development. Perhaps it is a universal mechanism for almost any types of cancer cells.
*It is possible that other cancers show some similarities in cancer metabolism because the importance of one carbon and methyl cycle is not exclusive to glioblastoma. However, very recently, one carbon metabolism has been highlighted as an important pathway in glioblastoma proliferation. This is in line with the relevance of one carbon intermediates in mediating processes directed related to generation of redox cofactors, polyamine synthesis, and purine synthesis, amongst other necessary processes. (JL May, et al. Sci Adv., doi: 10.1126/sciadv.aat0456 [2019]. PMID: 30613765; R Zgheib, et al. Cell Death Dis, doi: 10.1038/s41419-019-1836-2 [2019]. PMID: 31395852; C Ye, et al. Trends Endocrinol Metab., doi: 10.1016/j.tem.2018.06.002 [2018]. PMID: 30001904.)*

8.No supplementary Figure 5 and supplementary Figure 6 were found.
*We do not understand why Reviewer 1 did not see these figures. They were definitely uploaded and we have not been contacted regarding problems in Figure S5 and 6.*

9.While this study initially analyzed the tissue-specific expression of SERBP1, it lacks a genome-wide screening or identification process of this protein, which somewhat makes this study fall a little bit out of Genome Biology publication scope.
*We completely disagree. Our article focuses on glioblastoma. It has never been our intention to analyze SERBP1 function in normal tissue. We have never mention “tissue-specific expression of SERBP1”. In Figure 1, we show SERBP1 expression in multiple tissues and highlight the low expression levels in normal brain. This is important to show the relevance of SERBP1 increased expression in GBM and later, its role as an antagonist in neuronal differentiation. The comment “lacks a genome-wide wide screening or identification process of this protein” does not make much sense and it is unclear what the reviewer is looking for.
We and others published articles in Genome Biology focusing on genomic analyses of RNA binding proteins. Our study has a strong genomics component that includes RNA-seq, RIP-seq, RNAcompete, expression analysis using TCGA and GTex data and metabolomics. In particular, our study is a good fit for Genome Biology since establishes via genomic analysis a new function for an oncogenic RNA binding protein and its impact on epigenetic regulation via its role in metabolism.*

Reviewer #2
1) Did the authors perform an unbiased principal component analysis of their metabolomic data? Clearly there is a lot of metabolic data in their supplementary material and I feel the quality of the manuscript will be improved if an unbiased analysis is done.
*An unbiased principal component analysis was performed and both PCA scores plot and loadings plots were included in the revised manuscript as Figure S4A and B. Also a volcano plot was included Figure S4C.*

2) Did the authors examine any patient-derived GBM models? The U251 and U343 lines have been in culture for a long time and are poor models of the in vivo disease.
*In addition to the GBM cell lines, we analyzed the impact of SERBP1 KD on proliferation and survival of five GSCs (three mesenchymal (3565, 83NS, 1123NS), and two proneural (19NS, 84NS)). Differentiation and xenograft experiments were also conducted using GSCs.*

3) Did the authors measure glutathione levels by any complementary methods other than their mass spectrometry analysis? They show that cysteine levels are significantly reduced in shSERBP1 cells, and claim that "since cysteine is a precursor of glutathione (GSH), a redox regulating metabolite, its depletion might affect the redox balance in SERPB1 knockdown cells (Fig. 5B).". This is an important point- however I don't see an alteration in glutathione levels in Supplementary table 7.
*Glutathione levels were measured by LC-MS analysis and metabolite identification was confirmed using our internal library of standards. In the revised manuscript, we included intracellular glutathione content and conducted additional measurements using the GSH/GSSG-Glo Assay (Promega) as described in our previous publications (e.g. S. Kshattry, et al. Npj Precision Oncology, doi: 10.1038/s41698-019-0088-z [2019]. PMID: 31231686; S. L. Cramer, et al. Nature Medicine, 23, 120-127, PMID: 27869804 [2017]).*

4) Can the authors explain why there is no difference in cystathionine levels following SERBP1 silencing? Since homocysteine levels are altered, I would have expected to see a difference in cystathionine levels.
*We fully agree with the reviewer, and we were expecting a difference in cystathionine levels as well. It is also true that we performed an untargeted metabolomic analysis that can only provide an overall metabolic total pool trend. To underpin the cystathionine level, we are considering for future experiments to perform metabolic flux analysis (MFA) using multiple stable isotope 13C tracers. MFA will help elucidate not only the total pool of cystathionine, but also each contributing fraction of cystathionine that stems from multiple metabolic pathways.*

5) The logic behind investigating the PI3K/Akt pathway in SERBP1 knockdown cells is not clear. There are other pathways that control the GBM epigenome. Can the authors please clarify?
*The reason we selected the PI3K/Akt pathway for further studies was that the Gene Ontology analysis indicated that this pathway was enriched among genes downregulated in SERBP1 knockdown cells.*

6) The authors state that "SERBP1 OE cells also had increased mitochondrial respiration and ATP production compared to controls (Additional File 1: Fig. S8D-E). But I didn't see a clear difference in ATP levels following SERBP1 knockdown (Supp table 7). Can the authors explain the disconnect?
*The metabolomic study utilized cells stably expressing a tet-inducible knockdown system, whereas the OCR data was collected with cells stably overexpressing SERBP1. Overexpressing cells (pUltra vs pUltra-SERBP1) had been generated over several weeks, before OCR data was collected, while metabolomic data was collected 24 hours after induction of SERBP1 shRNA. The two cell systems modulate SERBP1 in different ways, one acutely (inducible knockdown), while the other chronically (long-term SERBP1 overexpression), which explains the disconnect in the two assays. Timepoints varied due to optimization of each protocol’s methods to collect the best quality data.*

**Second round of review**

**Reviewer 2**

The authors have thoroughly and clearly addressed all my comments for the original manuscript. I have no further questions and recommend that the manuscript be accepted for publication in the current format.
